# New implementation of data standards for AI in oncology: Experience from the EuCanImage project

**DOI:** 10.1093/gigascience/giae101

**Published:** 2025-05-13

**Authors:** Teresa García-Lezana, Maciej Bobowicz, Santiago Frid, Michael Rutherford, Mikel Recuero, Katrine Riklund, Aldar Cabrelles, Marlena Rygusik, Lauren Fromont, Roberto Francischello, Emanuele Neri, Salvador Capella, Arcadi Navarro, Fred Prior, Jonathan Bona, Pilar Nicolas, Martijn P A Starmans, Karim Lekadir, Jordi Rambla

**Affiliations:** Centre for Genomic Regulation (CRG), The Barcelona Institute of Science and Technology, Barcelona 08003, Spain; 2nd Department of Radiology, Medical University of Gdansk, 80-214 Gdansk, Poland; Clinical Informatics Service, Hospital Clínic de Barcelona, 08036 Barcelona, Spain; Department of Biomedical Informatics, University of Arkansas for Medical Sciences, 72205 Little Rock, Arkansas, United States; Social and Legal Sciences Applied to the New Technosciences Research Group, University of the Basque Country (UPV/EHU), 48940 Bilbao, Spain; Department of DIagnostics and Intervention, Diagnostic Radiology, Umeå university, 90187 Umeå, Sweden; Centre for Genomic Regulation (CRG), The Barcelona Institute of Science and Technology, Barcelona 08003, Spain; 2nd Department of Radiology, Medical University of Gdansk, 80-214 Gdansk, Poland; Centre for Genomic Regulation (CRG), The Barcelona Institute of Science and Technology, Barcelona 08003, Spain; Academic Radiology, Department of Translational Research, University of Pisa, 56126 Pisa, Italy; Academic Radiology, Department of Translational Research, University of Pisa, 56126 Pisa, Italy; Barcelona Supercomputing Center (BSC), 08034 Barcelona, Spain; Centre for Genomic Regulation (CRG), The Barcelona Institute of Science and Technology, Barcelona 08003, Spain; BarcelonaBeta Brain Research Center (BBRC), Pasqual Maragall Foundation, Barcelona, Spain; Universitat Pompeu Fabra (UPF), 08005 Barcelona, Spain; Institució Catalana de Recerca i Estudis Avançats (ICREA), 08010 Barcelona, Spain; Department of Biomedical Informatics, University of Arkansas for Medical Sciences, 72205 Little Rock, Arkansas, United States; Department of Biomedical Informatics, University of Arkansas for Medical Sciences, 72205 Little Rock, Arkansas, United States; Social and Legal Sciences Applied to the New Technosciences Research Group, University of the Basque Country (UPV/EHU), 48940 Bilbao, Spain; Department of Radiology and Nuclear Medicine and Department of Pathology, Erasmus MC Medical Center, 3015 GD Rotterdam, the Netherlands; Institució Catalana de Recerca i Estudis Avançats (ICREA), 08010 Barcelona, Spain; Artificial Intelligence in Medicine Lab (BCN-AIM), Departament de Matemàtiques i Informàtica, Universitat de Barcelona, 08007 Barcelona, Spain; Centre for Genomic Regulation (CRG), The Barcelona Institute of Science and Technology, Barcelona 08003, Spain; Universitat Pompeu Fabra (UPF), 08005 Barcelona, Spain

**Keywords:** interoperability, data model, artificial intelligence, FHIR

## Abstract

**Background:**

An unprecedented amount of personal health data, with the potential to revolutionize precision medicine, is generated at health care institutions worldwide. The exploitation of such data using artificial intelligence (AI) relies on the ability to combine heterogeneous, multicentric, multimodal, and multiparametric data, as well as thoughtful representation of knowledge and data availability. Despite these possibilities, significant methodological challenges and ethicolegal constraints still impede the real-world implementation of data models.

**Technical details:**

The EuCanImage is an international consortium aimed at developing AI algorithms for precision medicine in oncology and enabling secondary use of the data based on necessary ethical approvals. The use of well-defined clinical data standards to allow interoperability was a central element within the initiative. The consortium is focused on 3 different cancer types and addresses 7 unmet clinical needs. We have conceived and implemented an innovative process to capture clinical data from hospitals, transform it into the newly developed EuCanImage data models, and then store the standardized data in permanent repositories. This new workflow combines recognized software (REDCap for data capture), data standards (FHIR for data structuring), and an existing repository (EGA for permanent data storage and sharing), with newly developed custom tools for data transformation and quality control purposes (ETL pipeline, QC scripts) to complement the gaps.

**Conclusion:**

This article synthesizes our experience and procedures for health care data interoperability, standardization, and reproducibility.

## Background

Artificial intelligence (AI) for oncology is an exponentially growing field [1] built over large amounts of patient-related data. The volume and depth of personal health data necessary to support precision medicine are unprecedented, and the integration and analysis of such heterogeneous data types require a thoughtfully structured representation of knowledge [2, 3]. Besides, data need to be shared across diverse institutions and even across multiple nations, increasing the complexity of data integration and flows. FAIR principles (Findability, Accessibility, Interoperability, and Reusability) are the international reference that defines the best practices for data sharing. “Findability” entails the automatic discovery of datasets and services. “Accessibility” references the retrieval of the data, possibly including an approval process. “Interoperability” refers to the use of standards for data integration. Finally, “Reusability” implies an adequate description of the data (metadata) to optimize their use [4]. Adherence to the FAIR principles is imperative, and its application in health science and oncology is crucial for developing data sharing platforms [5].

Personal health data include a wide range of different data types, among others, demographic characteristics, the patient’s symptoms, diagnoses, laboratory results, medications, imaging data, and genomics. Generating and collecting these data types involves a multitude of different technological platforms (e.g., different vendors) and their storage in a wide range of data formats and information systems used by the different health care facilities, contributing to increased data diversity [6]. In fact, health care data have been shown to be more heterogeneous than other types of research data [7]. This high data complexity makes interoperability of health care information a significant hurdle in the development of AI models [8, 9]. The barrier is even more prominent in oncological observational research since cancer diagnoses require a set of attributes usually registered separately (imaging, histology, topology, grade, stage, and biomarkers), and the complex patient trajectory involves personalized treatment regimens [9].

Data need to be harmonized at 3 levels to address the interoperability challenge: technical, syntactic, and semantic. On one hand, requirements in technical interoperability facilitate basic data exchange conventions (file formats), and on the other hand, syntactic and semantic interoperability define data structure and the use of ontologies for unambiguous representation of medical concepts, respectively [10]. Across the different health care ecosystems, equivalent information can be represented in diverse ways. The use of standards that can be universally interpreted, both human and machine-readable, facilitates harmonization efforts. The structured exchange of health-related data is supported by international standards, such as the Fast Healthcare Interoperability Resources (FHIR) specification developed by Health Level Seven (HL7) international. FHIR defines the structure of medical data in modular components called “Resources” [11]. It is envisioned that the FHIR framework can become critical for implementing AI technologies in the health sector, just as Digital Imaging and Communication in Medicine (DICOM) or Picture Archiving and Communication System (PACS) for imaging data [6].

The EuCanImage initiative is a European Council Research and Innovation Action–funded research project that comprises multidisciplinary teams with the overall aim of building a data-sharing platform to be filled with over 20,000 cancer cases and AI models integrating imaging, clinical, and phenotypic data from 5 different EU countries to improve the outcomes of patients with cancer. Briefly, the EuCanImage platform integrates established data infrastructures: Collective Minds Radiology (CMRAD) platform for collaborative image annotation, the Eurobioimaging for image storage, the European Genome-Phenome Archive (EGA) for clinical and phenotypic data storage, and the Open EBench platform for AI algorithm benchmarking [12]. In this report, we synthesize our experiences as an overview of the methods and challenges we identified while working toward the standardization and interoperability of health data (clinical data) and implementing a data model for AI in large-scale oncology research.

## Data Collection

### Purpose for data collection and data description

EuCanImage is a complex project centered on addressing 7 key unmet clinical needs in cancer imaging [12–14]. A multidisciplinary team of different specialty physicians (radiologists, oncologists, radiotherapists, surgical oncologists, pathologists), sociologists, psychologists, AI developers, data scientists, small and medium enterprises (SMEs), imaging and oncology research associations, and patients’ organizations collaborated to identify and refine core research questions. The consortium pinpointed the most urgent topics in liver, colon, and breast cancer and designed 7 clinical use cases to respond to each specific clinical need. More specifically, we concentrated our efforts on 1 use case on hepatocellular carcinoma addressing the detection of indeterminate small lesions, 3 on colorectal cancer (1 aimed to identify liver metastasis from pre- and postoperative computed tomography [CT] in patients with colorectal cancer and 2 on rectal cancer to identify lymph node metastasis in contrast-enhanced rectal magnetic resonance imaging [MRI] and predict the response to neoadjuvant treatment), and 3 on breast cancer to (i) identify patients likely to achieve pathological complete response to de-escalate neoadjuvant systemic therapy based on the single-point, pretreatment contrast-enhanced MRI; (ii) automatically differentiate benign and malignant lesions in screening mammograms; and (iii) distinguish molecular subtypes of breast cancer based on digital mammograms. Our ambition is to integrate clinical, pathological, and genetic data (nonimaging data) and radiological images (imaging data) to build algorithms going beyond standard practice, allowing personalized approaches informed by the best-quality data. The initial effort to obtain such high-quality data was dedicated to defining clinical consensus and requirements for the use cases with specifications of clinical data variables.

The mentioned factors necessitate a comprehensive data model incorporating multifactorial inputs from multiple data sources. In EuCanImage, data are submitted from 6 university hospitals in Italy, Lithuania, Poland, Spain (2 sites), and Sweden; national registries; and 2 research institutions from the Netherlands. Each of the centers uses its own imaging infrastructure and PACS as well as electronic or paper health records that include demographic, clinical, pathological, and phenotyping information recorded in health information systems.

**Figure 1:**
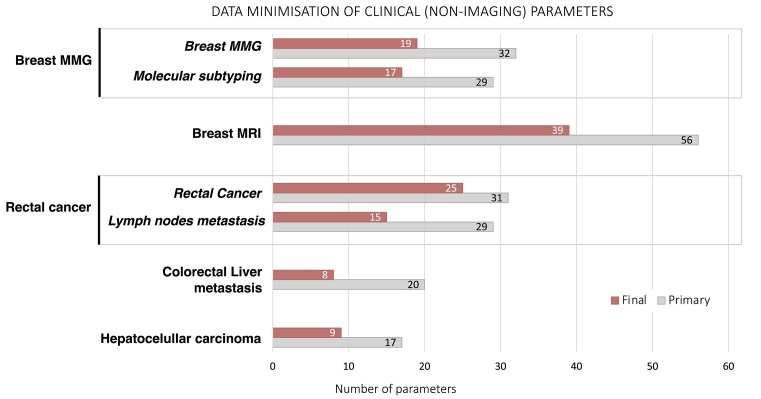
Data minimization of clinical (nonimaging) parameters.

Regarding the clinical data defined for each use, some common variables exist for all use cases: patient ID, biological sex, age at diagnosis, diagnosis, and pathology (ICD-O-3 codes). On the other hand, there are use case–specific variables such as the hormone receptor status, HER2 mutational status, Ki67 status for breast cancer, or information on specific chemotherapy agents with dosing regimens. The dialogue between physicians and AI developers on clinically relevant variables that can be meaningfully incorporated in AI algorithms, with the General Data Protection Regulation (GDPR)–compliant data minimization principle, led to the final set of defined variables (Fig. 1).

The selection of the clinical variables was a complex and lengthy procedure starting at the beginning of the EuCanImage project with the creation of the Clinical Working Group, including clinical representatives from all participating clinical centers. Each clinical site delegated a broad representation of specialists: radiologists, pathologists, clinical oncologists, surgical oncologists, radiotherapy specialists, and data managers. In general, the Clinical Working Group meetings were attended by 10–20 doctors every 2 weeks for the first year of the project. After general concepts of the use cases were established, the Clinical Working Group was divided into organ-related meetings (breast, colorectal, and liver subgroups) integrating different specialists per use case and center. These organ-related specialized groups met every 2 weeks for the next 6 months to develop the final list of clinicopathological variables using the Delphi consensus methodology. The final variables are deemed to provide both the ground truth and the clinical data with additional value for deep learning (DL) modeling.

The final number of clinical variables used as ground truth or additional input parameters for DL varies between 8 and 39 variables per use case. This clinical information will be used along with the information extracted from radiological images to build next-generation DL models combining both the clinical and the imaging information simultaneously. Three levels of data provision were defined: minimal, mandatory, and recommended. The minimal set contains essential information from the pathology assessment of specimens (e.g., cancer vs. other findings and the presence of complete pathological response vs. partial or no response). It allows the assembly of standard-level algorithms primarily using imaging information as input with pathology information as ground truth. The mandatory set contains important enriching information. These are all variables that should be included as input together with cancer images for more advanced and complex algorithms. Finally, the recommended set addresses additional clinical data points (e.g., risk factors for breast cancer) and phenotyping information (PAM50 results) available only from selected centers but with adequate numbers of patients for AI research. This recommended set would create a very interesting and promising asset in the project repository for future research that is otherwise not readily available from other data repositories.

Next, for each clinical variable, we defined comprehensive and detailed value sets to standardize concept representation and link the terms with ontological codes, ensuring unequivocal understanding. It allows good description of cohorts and, at the same time, prevents very fine-grained stratification of data with limited instances and unbalanced distribution in some of the cohorts. This consensus approach represents a compromise between the need for a precise representation of the clinical range of disease presentations and the goals of data clarity and homogeneity.

## Data Curation

### Analysis of semantic interoperability and health standards

Semantic interoperability represents a remarkable challenge for medical research. Data captured through health information systems are usually stored in locally modeled clinical repositories, mostly in nonstructured ways, thus hindering cross-national data source integration and translational research. Health information standards play a crucial role in defining the structure and meaning of clinical information so that different systems can unequivocally interpret it. However, there is no single standard that solves every need in the biomedical field; rather, there are different standards that complement or compete with one another. This includes standardized vocabularies and classifications and also health information standards. Examples of vocabularies include the Systematized Nomenclature of Medicine—Clinical Terms (SNOMED CT) [15] or International Classification of Diseases (ICD) [16], Logical Observation Identifiers Names and Codes (LOINC) [17], OHDSI Standardised Vocabularies, and International Cancer Genome Consortium [18]—Accelerating Research in Genomic Oncology (ICGC ARGO [19]). Examples of health information standards include HL7 FHIR [20] and open Electronic Health Records (openEHR) [21].

Vocabularies and classifications represent concepts that pertain to the biomedical domain in a standard fashion [22], although they require a common structure that provides the syntactic interoperability required to achieve semantic interoperability. Common Data Models (CDMs) serve as representations of collected data aimed at facilitating the exchange, pooling, sharing, or storing data from multiple sources and can provide this common structure [23]. Health information standards also provide a syntactic base to allow the formal representation of the structure of clinical information and its meaning.

FHIR was introduced in 2011 by the standard-developing organization HL7 [20]. The information within FHIR is organized in basic building blocks named Resources. Those blocks define the structure of the contained information. Although it is widely used in health informatics, its uptake in research environments is less prevalent [24]. Most studies using FHIR in health research focus on clinical research (including clinical trials), and just about 12% are oncology related [25]. In these studies, FHIR has been mainly used for standardization and data capture and, to a lesser extent, for data analysis [25].

Observational Medical Outcomes Partnership (OMOP) enables the systematic analysis of disparate observational databases through a common data model and a closed dictionary of terminologies, vocabularies, and coding schemes. Several authors consider it an adequate data model for sharing data in electronic health record (EHR)–based longitudinal studies [23, 26].

ICGC ARGO [19] is an initiative that provides a fixed schema for creating 15 clinical tables oriented to genomic oncology research, thus oriented for addressing cancer-specific issues in the representation of clinical data.

### Data model design

Data standardization within the project is required to (i) support content organization and subsequent development of AI algorithms and facilitate (ii) interoperability and (iii) the secondary use of the data (i.e., data distribution under request in a repository). Both OMOP and FHIR are widely adopted standards in clinical settings; however, they were conceived to serve different purposes. OMOP is more oriented toward clinical data representation (structure and content), and FHIR is more focused on health care data exchange. After thoroughly evaluating various CDM alternatives, we decided to use FHIR due to its wide adoption, flexibility, and suitability for real-world data exchange. More importantly, and the key aspect we considered in selecting FHIR over OMOP, was its appropriateness for permanent data storage and long-term data sharing through the repository.

**Figure 2:**
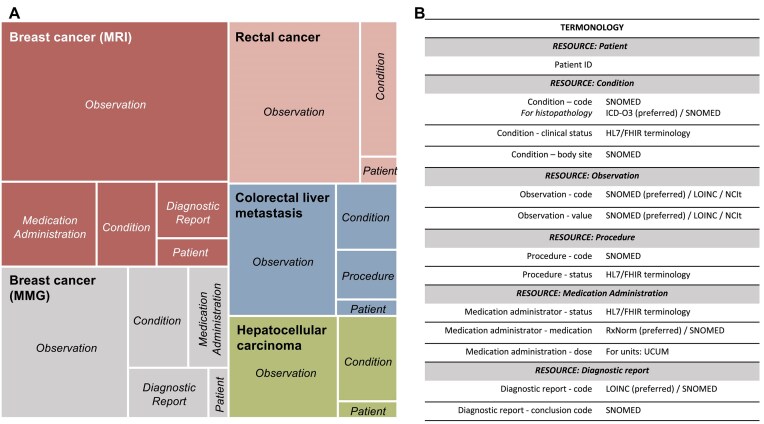
(A) Representation of the proportion of FHIR resources needed for each use case. (B) Ontologies used in each resource.

**Figure 3:**
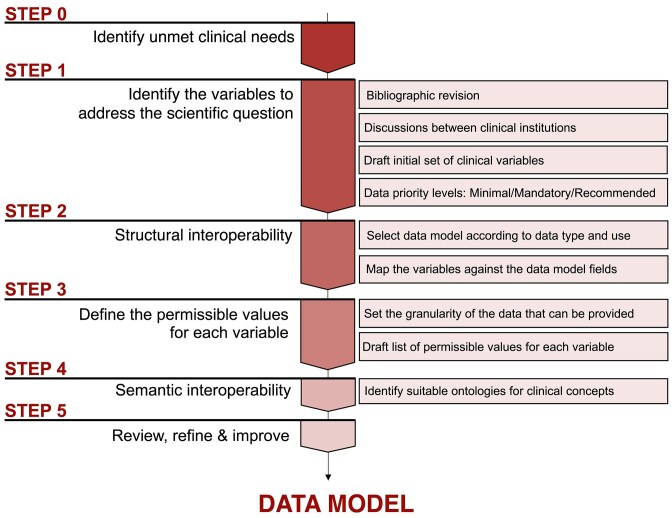
Description of the different steps followed to conceptualize the data model.

As previously outlined, clinical elements necessary for each hypothesis were established by domain experts and interdisciplinary teams, including clinicians and AI developers, who considered different key data aspects. Some key considerations for variable selection were characterization of the target population, clinical endpoints (pathological hallmarks, disease behavior, treatment response, and the patient prognosis), type of outcome (binary, continuous, time to event), adequate ground truth, minimal amount of data principle, and the availability of specific variables at data sources. For the project, data to cover the 7 use cases were arranged in 5 different data schemas with single schemas used by multiple use cases. As a general overview, the highest level components of the FHIR model are the Resources, which contain hierarchical sublayers of descriptive elements for more detailed data classification. The content and format of a Resource have controlled properties, meaning that the different data elements and data types need to adhere to specific requirements. To design the data architecture needed for each EuCanImage use case, the following FHIR resources were identified as relevant: Patient, Condition, Observation, Procedure, Medication Administration, and Diagnostic report (Fig. 2A). When choosing the most suitable resource for each selected variable, the resource’s constraints were considered. For example, the classification of patients into case (cancer) or control (benign lesion) groups could be interpreted as part of the Condition Resource, alternativelly, information about a diagnosis can be stored in an Observation Resource, capturing the results of tests (mammogram). In this example, we considered it within the Condition Resource despite also including benign cases, so it could be associated with age at diagnosis. Once the variables were assigned to a Resource, they were mapped to the appropriate FHIR element. In cases where the clinical variables could be assigned to more than 1 suitable profile (e.g., histological type), simplicity criteria were applied to minimize the number of Resources used. Many data elements within the FHIR Resources require coded values. Some are fixed values defined by the FHIR specification, but others require external ontologies. As a general rule, HL7/FHIR terminology was used in a few established fields, specifically status profiles. SNOMED was the preferred terminology for general clinical concepts, ICD-O3 for histology, LOINC for some test observations, and RxNORM for medication. We used NCIT when the concept did not exist in previous ontologies (Fig. 2B). The summary of the different stages we followed to conceptualize the data model is described in Fig. 3.

It is essential to point out that the project presented some particular needs that were not fully represented by FHIR (and standards ontologies), requiring alternative solutions to overcome limitations. The gaps identified relate to the fact that FHIR was designed to support interoperability and data exchange in health care rather than specifically focusing on research needs. The primary limitations we faced were (i) the need to represent concepts without available standard terminology, (ii) variables not structured as in health care practice, (iii) the representation of dates to comply with the deidentification of personal data, (iv) the representation of not provided (missing) information, and (v) the implementation of the model without a FHIR server.

For each clinical variable, we defined the limited set of permissible values (value set) that this variable can adopt. Some of these value sets needed to include ambiguous concepts for simplicity and data harmonization reasons. An example is the term “other,” which is required to group less frequent or more irrelevant values. Those terms, isolated from additional context, posed a challenge for interoperability. We used SNOMED post-coordinated expressions to build more specific clinical ideas by combining relevant terms with compositional grammar. Another challenge posed was concepts that are not used in health care but are essential to contextualize the specific use cases for research purposes and that are not captured by standard terminologies. Some examples are the variable “breast cancer subtype by proxy” to group patients with breast cancer according to hormone receptor, Ki67, and HER2 expression levels or “time interval between the end of the neoadjuvant treatment and surgery.” Additional difficulties include tumor grading systems such as the modified Ryan Scheme for Tumor Regression Grade, Miller and Payne’s Tumor Regression Grade, Residual Cancer Burden class, or grading of Ductal carcinoma in situ (DCIS). Our approach was using the specific grading scales in NCIT, if available, or using generic grading scales in SNOMED (e.g., 1 on a scale of 1 to 5), despite the fact it could affect interoperability.

Some variables were characterized under the Medication Administration Resource that presented significant difficulties in creating the representations needed for the project. For example, to detail the chemotherapy dosage, we required the total number of chemotherapy cycles, dose (amount of medication per dose), and the accumulation dose within the same “Medication administration” entry. However, that Resource is designed to collect only a single entry.

In compliance with GDPR, personal data pseudonymization entails the removal of indirect data identifiers, such as dates. The collection of exact dates was replaced by the collection time intervals (months, weeks, etc.). Most FHIR Resources allow time periods as valid data types, but the Medication Administration Resource only allows dates (*ddmmyy*). To fulfill this FHIR restriction, we recodified the time periods into arbitrary dates starting on 1 January 1970 to mimic the Epoch Unix system, with the end date calculated based on the collected time interval and starting date in mind.

## Technical Implementation of Data Standards

Transforming and loading “raw” data from various hospital data systems into the newly developed data schema proved to be challenging and labor-intensive. The harmonization efforts required by the different participating institutions and different use cases varied significantly depending on the existing resources at the sites. While some centers housed structured repositories with variables linked to standard terminologies that required minimal mapping and transformation efforts, others mapped local concepts with the project schema manually. This effort was performed by trained site personnel who understood the clinical concepts in both English and the center’s local language. Online support was provided by the EGA when needed.

The FHIR implementation format has a hierarchical architecture that, while having many advantages to encode the relationships between the variables and facilitating data storage, supposes an additional barrier for data providers given that most of the required information was not structured data inside their health records. To minimize the need to re-encode and simplify the data capture process, we created electronic case report forms (eCRFs) with REDCap. REDCap is a secure web application that supports data capture primarily for research studies [27, 28]. This software allows the custom design of data entry forms and data collection workflows. It features a user-friendly interface for designing the forms, field validation, custom logic patterns, calculated fields, data import/export options, data quality control, and role-based user access. Additionally, it offers a set of APIs for integration with other platforms [29]. REDCap was deployed at the EGA to design and manage data entry forms for clinical data collection within the consortium. Different data entry forms were conceived to support each of the 5 different data schemas.

Data from hospitals can be imported to the EuCanImage REDCap database following 2 paths: (i) by directly filling the online forms or (ii) by entering data into CSV files complying with specific REDCap format requirements and uploading the files into REDCap.

Patient IDs were previously pseudo-anonymized at the hospitals, and only hashed patient IDs (EuCanImage ID) were introduced in the platform. Consequently, all related clinical data from the different institutions merged into a single harmonized database for each use case. Once harmonized data were in the database, quality control checks were performed. All data were then exported from REDCap as a CSV file for conversion into FHIR-compliant files.

To implement the FHIR model, we created each Resource using individual persistent identifiers with Uniform Resource Names (URNs), more specifically with Universally Unique Identifiers (UUIDs). These identifiers were generated for each patient, resource, and bundle. In FHIR, a bundle is a way to gather all the Resources belonging to a single patient. In our case, a bundle is generated from a single row of the exported CSV files.

For the subsequent data standardization stage, we built Extract Transform Load (ETL) pipelines to transform the output CSV files into JSON files compliant with the FHIR schema.

**Figure 4:**
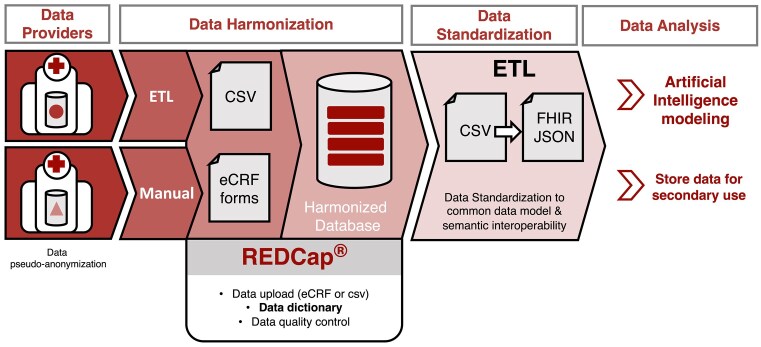
Clinical data processing workflow from clinical institutions to data analysis/sharing. Hospitals import data to REDCap following different paths depending on local resources: some centers introduce data manually (filling online forms or CSV files) or develop their own ETL scripts to automate the process. As a result, all clinical data from the different institutions are merged into a single harmonized database for each use case. Finally, all data are exported from REDCap as a CSV file for standardization and conversion into FHIR-compliant files (FHIR JSON) and stored at the EGA.

To automate this process, we used Python 3.11 and followed a FHIR 4.3 schema. The Python scripts are available on GitHub (see “Availability of Supporting Source Code and Requirements” section), with the additional use of external validators, such as FHIR Validator GUI [30] and Simplifier [31]. The methods section provides a more detailed description of the steps followed for creating the ETL scripts. While building the Python scripts, the mapping of the dictionaries was coded using FHIR-compliant ontologies. The results of the ETL process are JSON files containing the patients’ information standardized to the CDM, 1 file per patient. These files will serve both as the data source for AI algorithm development and, with proper data requests, standardized data available for the scientific community (Fig. 4).

### Data quality and consistency

Quality data can be defined as data that are fit for purpose (e.g., the data are sufficient for the specified purpose for which it is intended) [32, 33]. In most cases, data quality for the purpose of machine learning cannot be limited to a single focus but must cater to the needs of multiple audiences. Data quality issues can be introduced at any point in the data management and collection life cycle. Whether during data acquisition, storage, analysis, or publication, diminished quality can inadvertently affect downstream tasks such as AI training [32–34].

We employ both quality assurance and quality control techniques over the course of the data life cycle, including strict conformance to requirements during input and the assessment methods for repeatable feedback and improvement. The overall objective of our quality analysis is to rate the individual records based on multiple dimensions of quality and use this as a filter for downstream tasks. To achieve the measure of data quality needed for superior AI training and results, we defined quality control rules and procedures based on standard dimensions of quality and built tools to integrate into our pipelines for data collection and storage.

Since we use REDCap as the intermediary data store where all collection methods funnel their data, we found REDCap’s data quality module [27, 28] valuable for organizing our data quality rules. This module allows for the execution of quality checks for all data entered into the system, whether by direct entry or by CSV imports. This also enabled the capability to export these rules for use in customized tools for data collection.

Data quality can be evaluated over many different dimensions [33], and we have focused our evaluation on 3 critical dimensions: completeness, conformance, and plausibility. For completeness, we focused on value requirements. For conformance, we analyzed the various data types and permissible values to ensure adherence. Plausibility applied to ranges, such as age. The types and dimensions are outlined in Table 1.

**Table 1:** Clinical data quality assessment dimensions

Type	Dimension	Description
Minimal_req	Completeness	Meets minimum requirements
Mandatory_req	Completeness	Meets mandatory requirements
Length	Conformance	Conforms to length restrictions
Datatype	Conformance	Conforms to data type restrictions
Permissible	Conformance	Conforms to list of permissible values
Range	Plausibility	Meets known range limits

Much of the needed data quality assessment functionality was already built into the REDCap quality module, including preestablished rules handling blank values, data type errors, outliers, and invalid permissible values. We also included custom rules covering multiple levels of requirements: minimal, mandatory, and recommended. These rules aligned with the required fields outlined for each use case and agreed upon by representatives from each clinical center.

After assessment of the data based on these organizing quality criteria, we generate a score for each quality check based on the number of successes and failures. Here, we can also apply weights if we deem a particular assessment more important than others. Table 2 shows an example of a scoring report.

**Table 2:** Data quality scoring report

	Dimension	Type	Fail	Pass	Total	Weight	Score
0	Completeness	minimal_req	2	16	18	50	44.44%
1	Completeness	mandatory_req	3	32	35	10	9.14%
2	Conformance	length	1	10	11	10	9.09%
3	Conformance	datatype	0	40	40	10	10.00%
4	Conformance	permissible	3	52	55	10	9.45%
5	Plausibility	range	1	3	4	10	7.50%
**Total:**			**10**	**153**	**163**	**100**	**89.63%**

## Legal Aspects

In EuCanImage, ethicolegal discussions have played an important role from the beginning of the project and continue to be a recurring topic in similar consortia and initiatives. In addition to the well-known General Data Protection Regulation (GDPR), the past decade has witnessed a surge in regulations and norms that are directly or indirectly relevant when trying to implement data standards for data interoperability. These include, among others, the recently passed Data Governance Act (DGA), the Artificial Intelligence Act (AIA), and the proposal for a regulation on the European Health Data Space (EHDS). Such regulatory developments are likely to be of considerable practical relevance to the scope of this article, in particular, for discussions on the personal or nonpersonal nature of datasets involved, legal barriers for secondary uses of data for AI research, and development and cross-border data sharing for AI research in oncology.

### Personal data vs. nonpersonal data

As anticipated, early discussions within research projects and consortia often concern the personal or nonpersonal nature of the data to be processed. This is mainly due to the fact that processing operations involving personal data—namely, information relating to an identified or identifiable natural person—fall under the scope of the GDPR. Therefore, nonpersonal data such as anonymized data (i.e., personal data rendered anonymous in such a manner that the data subject is not or no longer identifiable) are not bound by the regulation. Pseudonymized data, which could be attributed to a natural person by the use of additional information, would qualify as personal data (see Article 4(5) and Recital 26 of the GDPR [35]). Generally, deidentification, understood as the process of removing or substituting all personal information and identifiers, is not sufficient to achieve the anonymization threshold required in the EU. As a result, if the raw data are retained at the source and any key or additional information can be used to reverse the process and reidentify the data subject, this information shall be considered pseudonymized data and thus subject to the GDPR.

Within EuCanImage, all direct and indirect personal identifiers have been removed. In addition, the data are double-hashed both by the data providers and by the platform. Ultimately, all processing operations within the project are aligned with the GDPR and supported by a contractual and governance structure, enabling compliant data sharing.

### Secondary use of data for AI research in precision medicine

Further processing of personal data for scientific research purposes, which comprises AI research in precision medicine as pursued by EuCanImage, is compatible with the GDPR (art. 5(1)(b)). Moreover, under the forthcoming EHDS regulation, secondary use of pseudonymized electronic health data is expressly permitted for “training, testing and evaluating of algorithms, including in medical devices, in vitro diagnostic medical devices, AI systems and digital health applications” (art. 34(1)(e)). It should be noted, nonetheless, that these 2 terms (“further processing” and “secondary use”) are not legally analogous, but the latter will be preferred here for the sake of clarity.

### Cross-border data sharing for AI research in precision medicine

Despite the advent of the GDPR and the harmonization effort, processing operations involving multiple organizations and researchers from several EU member states still face slight differences between national, regional, and sectoral regulatory frameworks. Hence, although data sharing within member states of the European Economic Area (EEA) is not hindered by any additional requirements, EuCanImage’s partners and their legal teams still must cope with a complex and fragmented scenario not only from a legal interoperability perspective but also concerning divergent ethical oversight layers and internal procedures at each center, hospital, institution, or country.

Transfers of personal data to third countries or international organizations (i.e., data sharing with researchers or organizations outside the EEA) remain controversial [36, 37]. Even though EuCanImage members do not plan to store or process data outside the EEA, controversies have arisen in relation to the transfer of data to international organizations and UK-based institutions after Brexit. Potential routes for transfers remain limited, particularly in light of the strict requirements and threshold set by the Court of Justice of the European Union [38, 39].

## Discussion

In many scientific disciplines, especially in health research, working with large-scale datasets and engaging in cross-border data sharing is becoming increasingly vital for the adoption and development of AI technologies. EuCanImage focuses on leveraging existing health care data to address various scientific research questions using AI models. Our experience uncovers obstacles to data interoperability and reuse, as well as realistic solutions. In this report, we outline our procedures to achieve data interoperability, including a thorough description of the data model design, the standards used, harmonization efforts, and methodological aspects concerning the practical implementation, along with legal interoperability considerations. The successful development and deployment of our data models and related standards represent a significant milestone, laying the groundwork for future AI applications in cancer health care. Furthermore, our work highlights the need for improvements in data collection, annotation, and cross-border dissemination.

We anticipate that our approach and methods will benefit individual institutions and serve as a guide for future large-scale consortia requiring harmonization and interoperability of cancer-related clinical data for AI and machine learning advancements. Similar efforts have been developed by other consortia [40], including projects within the AI4HI initiative such as CHAIMELEON, ProCancer-I, Incisive, and Primarge [14]. These collective endeavors involve meticulous, collaborative, expert-driven analysis, spanning model design, data curation, standards usage, and infrastructure development. The knowledge and experience gained from our combined efforts are crucial in laying the groundwork for future health care data standardization initiatives for AI research across Europe.

Achieving interoperability in health care data raises complex issues that need to be addressed. The development of supervised AI models trained for prediction or classification tasks relies on data labeled with “ground-truth” classifications. Reaching a consensus on data labeling requires common standard definitions for diagnosis and agreements on the level of data granularity; these are critical factors that affect the reproducibility and quality of the results [41, 42]. Cancer diagnosis involves integrating complex criteria based on a variety of disparate data components, such as pathology reports, laboratory results, radiology findings, and advanced molecular and genetic tests. Close collaboration among different medical specialists has enabled the establishment of key principles for data harmonization: (i) selecting and defining essential clinical variables to address medical needs, (ii) identifying common data available across all centers, and (iii) striking a balance between the volume and granularity of the data that can be provided by various hospitals and the optimal information required for AI models (Fig. 3).

Within the health care–research ecosystem, data sharing remains a barrier. Yet, it is a crucial mechanism for ensuring that high-quality data, obtained through exhaustive and expensive processes like defining data labels, harmonization tasks, and the use of common standards, can be reused by other researchers and, thus, maximize the impact. The FAIR principles provide the framework for such data reuse [43]. Despite progress in adopting interoperability standards, data from different sources still contain discrepancies. To make data fully reusable and reproducible, methods for data cleaning, harmonization, and standardization must be transparent [44].

While the presented work demonstrates the feasibility of using HL7 FHIR to achieve interoperability, it also has limitations. FHIR resources were employed for structural interoperability, while SNOMED, LOINC, NCIT, and RxNorm were mainly used for semantic interoperability. By leveraging the comprehensive information model in FHIR, clinical data can be organized hierarchically in a manner that captures its context and remains unambiguous [45]. However, utilizing FHIR to build a model for research oncology presents specific constraints and unique requirements for maintaining data interoperability (described previously in the data model section). To maximize the potential of FHIR and encourage broader adoption in the specialized scientific context of AI for precision medicine, alternative FHIR configurations or detailed methodological explanations should be considered to ensure reproducibility. Currently, the main limitation is that the suitability of the models for developing AI algorithms has not yet been validated.

In summary, we demonstrated that large-scale, real-world, multicenter clinical data harmonization and curation for AI research is feasible through the use or adaptation of common standards. The standardized datasets we will make available at the end of the project, including data from over 20,000 patients with cancer, will provide an invaluable resource for investigators to expand the understanding of these complex diseases and open the door for cutting-edge translational research beyond the scope of EuCanImage.

## Methods

### Data model

The clinical data necessary to address each use case were established by interdisciplinary teams, including clinicians and AI experts, considering different key data aspects. Data were arranged following 5 different data schemas that were compliant with the FHIR (FHIR Release 4B) architecture. The FHIR Resources used were Patient, Condition, Observation, Procedure, Medication Administration, and Diagnostic report.

### Ontologies

HL7/FHIR terminology was used in status profiles required by FHIR. SNOMED (SNOMED version International 2022–12-31) was the preferred terminology for general clinical concepts, ICD-O3 (ICD-O3 version 20220429) for histology, LOINC (LOINC version 2.73) for some test observations, and RxNorm (RxNorm version 3 January 2023) for medication. We used NCIT (NCIT version 23.8d) when the concept did not exist in previous ontologies.

### Data capture, standardization, and quality control

Patient IDs were pseudo-anonymized at the hospitals, and only hashed patient IDs (EuCanImage ID) were introduced in the platform. Data from hospitals were captured in REDCap (REDCap version 13.10.0; PHP 8.1.3 [Linux/Unix OS]; MySQL 8.0.30) by filling the online forms or uploading CSV files complying with the specific format requirements. As a result, all clinical data were merged into a single harmonized database for each data schema. At this stage, we performed quality control checks. We focused our evaluation on 3 critical dimensions—completeness, conformance, and plausibility—and generated a score for each quality check based on the number of successes and failures. We built ETL pipelines in Python to transform the harmonized output data into JSON files compliant with FHIR. We used FHIR Validator GUI [30] and Simplifier [31] as external validators for quality control. Code availability: The Python scripts to transform harmonized data to the FHIR compatible schemas are available on GitHub (see “Availability of Supporting Source Code and Requirements” section).

### Steps to create the ETL script

**Step 0—Dictionary:** Before processing the data, we set up an environment with the necessary resources. This includes the creation of a machine-readable data dictionary encoding (i) the name of the variable, (ii) the ontological code, and (iii) the RedCap internal codification. This mapping served 2 purposes simultaneously: (i) data quality control and (ii) the ETL process.
**Step 1—Parsing:** All the data gathered in REDCap were exported into a CSV file per use case and clinical center. These CSV files were parsed into a Python-readable form for the posterior transformation into the objects required by the libraries.
**Step 2—Dictionary import:** For the transformations to occur with minimal errors, dictionaries from step 0 were imported into the Python script for the consecutive mapping to the FHIR Resources.
**Step 3—FHIR Resource mapping:** To streamline the transformation of the different types of data variables into their respective FHIR Resources, we defined functions to automate the process.
  1. First, empty templates were created for each FHIR Resource type. To avoid errors, we maintained libraries that followed FHIR structures with internal validators.
  2. Then, depending on the input required by each function (such as information about medication administrations, quantitative or qualitative observations, conditions from the patients or others), the Resource was populated accordingly. Additionally, some variables needed extra processing, such as date and timestamp parsing, which is also automated by the code.
  3. In the case of an error in the structure, the libraries flag them for correction.
**Step 4—Export and validation:** As a final step, the objects that were created in the script needed to be exported into JSON files. Since the code included FHIR libraries streamlining the process, parsing the generated objects and dictionaries into the JSON file was straightforward. The libraries used in the process validate the integrity of the structure but not always of the contents. To confirm the correctness of the result, we used external validators, such as FHIR Validator GUI [30] and Simplifier [31].

### EGA

The EGA is a service for permanent archiving and sharing of personally identifiable genetic, phenotypic, and clinical data. The standardized clinical data, 1 JSON file (FHIR compliant) per patient obtained after the previously described process, will be encrypted and stored at the EGA repository.

## Availability of Supporting Source Code and Requirements

Project name: EuCanImage FHIR implementation

Project homepage: https://github.com/EGA-archive/EuCanImage-FHIR/

Operating system(s): Platform independent

Programming language: Python

Other requirements: Python 3.11.2 or higher, FHIR Resources 6.5.0 or higher, pandas 2.1.3 or higher, numpy 1.26.2 or higher

License: Apache License 2.0

RRID: SCR_025824

## Abbreviations

AI: artificial intelligence (AI); AIA: Artificial Intelligence Act; CDM: Common Data Model; CMRAD: Collective Minds Radiology; CT: computed tomography; DGA: Data Governance Act; DICOM: Digital Imaging and Communication in Medicine; DL: deep learning; eCRF: electronic case report form; EEA: European Economic Area; EGA: European Genome-Phenome Archive; EHDS: European Health Data Space; EHR: electronic health record; ETL: Extract Transform Load; FAIR: Findability, Accessibility, Interoperability and Reusability; FHIR: Fast Healthcare Interoperability Resources; GDPR: General Data Protection Regulation; ICD: International Classification of Diseases; ICGC ARGO: International Cancer Genome Consortium—Accelerating Research in Genomic Oncology; LOINC: Logical Observation Identifiers Names and Codes; MRI: magnetic resonance imaging; OMOP: Observational Medical Outcomes Partnership; openEHR: open Electronic Health Records; PACS: Picture Archiving and Communication System; SME: small and medium enterprise; SNOMED CT: Systematized Nomenclature of Medicine—Clinical Terms; URN: Uniform Resource Name; UUI: Universally Unique Identifier.

## EuCanImage CONSORTIUM (Lead Authors Extended List)

**Jordi Rambla—**Centre for Genomic Regulation (CRG), The Barcelona Institute of Science and Technology, Barcelona, Spain
**Maciej Bobowicz**–2nd Department of Radiology, Medical University of Gdansk, Gdansk, Poland
**Jordi Rimola and Xavier Bargalló—**Fundació Clínic per a la recerca biomedica, Barcelona, Spain
**Fred Prior**—Department of Biomedical Informatics, University of Arkansas for Medical Sciences, Little Rock, Arkansas, United States
**Pilar Nicolas**—Social and Legal Sciences Applied to the New Technosciences Research Group, University of the Basque Country (UPV/EHU), Bilbao, Spain
**Katrine Riklund**—Department of Diagnostics and Intervention, Diagnostic Radiology, Umeå University, Umeå, Sweden
**Lorenzo Faggioni—**Academic Radiology, Department of Translational Research, University of Pisa, Pisa, Italy
**Josep Lluis Gelpi—**Barcelona Supercomputing Center (BSC), Barcelona, Spain
**Martijn P. A. Starmans**—Department of Radiology and Nuclear Medicine and Department of Pathology, Erasmus MC Medical Center, Rotterdam, the Netherlands
**Kaisar Kushibar and Karim Lekadir**—Artificial Intelligence in Medicine Lab (BCN-AIM), Departament de Matemàtiques i Informàtica, Universitat de Barcelona, Barcelona, Spain
**Karim Lekadir—**Institució Catalana de Recerca i Estudis Avançats (ICREA), Barcelona, Spain
**Philippe Lambin and Henry Woodruff**—Universiteit Maastricht (UM), Maastricht, The Netherlands
**Melanie Goisauf**—Biobanks and Biomolecular Resources Research Infrastructure Consortium (BBMRI-ERIC), Graz, Austria
**Davide Zaccagnini—**Lynkeus (LYN), Rome, Italy
**Pär Kragsterman**—Collective Minds Radiology AB (CMRAD), Täby, Sweden
**Flore Belmans**—Oncoradiomics (ONCO), Liege, Belgium
**Tobias Heimann**—Siemens Healthcare GMBH (SIE), Erlangen, Germany
**Peter Gordebeke**—EIBIR Gemeinnutzige GMBH zur Forderung der Erforschung der Biomedizinischen Bildgebung (EIBIR), Wien, Austria
**Emanuele Neri—**European Society of Oncologic Imaging—ESOI Europaische Gesellschaftfur Onkologische Bildebung (ESOI), Vienna, Austria
**Jane Smith**—European Association for Cancer Research (EACR), Nottingham, UK
**Juozas Kupčinskas—**Lietuvos Sveikatos Mokslu Universiteto Ligonine KaunoKlinikos (KAUNO), Kaunas, Lithuania
**Javier del Riego**—Institut d'Investigació i Innovació Parc Taulí I3PT, Universitat Autònoma de Barcelona, Barcelona, Spain

## Supplementary Material

giae101_Authors_Response_To_Reviewer_Comments_Original_Submission

giae101_Authors_Response_To_Reviewer_Comments_Revision_1

giae101_Authors_Response_To_Reviewer_Comments_Revision_2

giae101_GIGA-D-24-00085_Original_Submission

giae101_GIGA-D-24-00085_Revision_1

giae101_GIGA-D-24-00085_Revision_2

giae101_GIGA-D-24-00085_Revision_3

giae101_Reviewer_1_Report_Original_Submission Andrey Fedorov -- 5/12/2024

giae101_Reviewer_1_Report_Revision_1 Andrey Fedorov -- 8/8/2024

giae101_Reviewer_2_Report_Original_Submission Charles Vesteghem -- 5/16/2024

giae101_Reviewer_2_Report_Revision_1 Charles Vesteghem -- 8/5/2024

## Data Availability

An archival copy of the code is available via Software Heritage [46] and a Workflowhub collection [47] with the following items: hepatocellular carcinoma [48], colorectal liver metastasis [49], rectal cancer [50], breast cancer MMG [51], and breast cancer MRI [52]. Since EuCanImage is still ongoing, the data are not available yet. The datasets will be available at the end of the project under controlled access at the European Genome-Phenome Archive (clinical data) and EuroBioimaging (images).
