# Supplementary material for: New implementation of data standards for AI in oncology: Experience from the EuCanImage project
Source: Gigascience. 2025 May 13;14:giae101. doi: 10.1093/gigascience/giae101 (PMC12071370; doi:10.1093/gigascience/giae101)

GigaScience

New implementation of data standards for AI in oncology. Experience from the EuCanImage project.

--Manuscript Draft--

**Manuscript Number:**	GIGA-D-24-00085R1
**Full Title:**	New implementation of data standards for AI in oncology. Experience from the EuCanImage project.
**Article Type:**	Technical Note
**Funding Information:**	H2020 European Institute of Innovation and Technology (952103) — Not applicable
**Abstract:**	Background: An unprecedented amount of personal health data, with the potential to revolutionise precision medicine, is generated at healthcare institutions worldwide. The exploitation of such data using artificial intelligence relies on the ability to combine heterogeneous, multicentric, multimodal and multiparametric data, as well as thoughtful representation of knowledge and data availability. Despite these possibilities, significant methodological challenges and ethico-legal constraints still impede the real-world implementation of data models. Technical details: The EuCanImage is an international consortium aimed at developing AI algorithms for precision medicine in oncology and enabling secondary use of the data based on necessary ethical approvals. The use of well-defined clinical data standards to allow interoperability was a central element within the initiative. The consortium is focused on three different cancer types and addresses seven unmet clinical needs. We have conceived and implemented an innovative process to capture clinical data from hospitals, transform it into the newly developed EuCanImage data models and then store the standardised data in permanent repositories. This new workflow combines recognized softwares (REDCap for data capture), data standards (FHIR for data structuring) and an existing repository (EGA for permanent data storage and sharing), with newly developed custom tools for data transformation and quality control purposes (ETL pipeline, QC scripts) to complement the gaps. Conclusion: This article synthesises our experience and procedures for healthcare data interoperability, standardisation and reproducibility.
**Corresponding Author:**	Teresa García-Lezana, PhD<br>Centre de Regulacio Genomica<br>Barcelona, SPAIN
**Corresponding Author Secondary Information:**	
**Corresponding Author's Institution:**	Centre de Regulacio Genomica
**Corresponding Author's Secondary Institution:**	
**First Author:**	Teresa García-Lezana, PhD
**First Author Secondary Information:**	
**Order of Authors:**	Teresa García-Lezana, PhD
	Maciej Bobowicz
	Santiago Frid
	Michael Rutherford
	Mikel Recuero
	Katrine Riklund
	Aldar Cabrelles
	Marlena Rygusik
	Lauren Fromont

Powered by Editorial Manager® and ProduXion Manager® from Aries Systems Corporation

	Roberto Francischello
	Emanuele Neri
	Salvador Capella
	Fred Prior
	Jonathan Bona
	Pilar Nicolas
	Martijn P. A. Starmans
	Karim Lekadir
	Jordi Rambla
**Order of Authors Secondary Information:**	
**Response to Reviewers:**	R1 - COMMENT 1<br>1. p.6: The authors mention "seven key unmet clinical needs in cancer imaging", but it is not clear what those refer to. It would be helpful to provide details about those needs, and provide more details about the data being collected for the individual applications. Also, if possible, please cite a publication that describes EuCanImage project.<br><br>Thank you for pointing this out. We have included a brief description of the use cases (page 6 - Data description) and project (page 5 - Background) for better understanding. Also in response to comment 3. We don't have a dedicated publication describing the project, however detailed information about EuCanImage can be found in our webpage (https://eucanimage.eu/#about-project) and in publications by the AI4HI network:<br>Kondylakis, H., Kalokyri, V., Sfakianakis, S. et al. Data infrastructures for AI in medical imaging: a report on the experiences of five EU projects. Eur Radiol Exp 7, 20 (2023). https://doi.org/10.1186/s41747-023-00336-x<br>Marti-Bonmati, L., Koh, DM., Riklund, K. et al. Considerations for artificial intelligence clinical impact in oncologic imaging: an AI4HI position paper. Insights Imaging 13, 89 (2022). https://doi.org/10.1186/s13244-022-01220-9<br>Kondylakis, H., Ciarrocchi, E., Cerda-Alberich, L. et al. Position of the AI for Health Imaging (AI4HI) network on metadata models for imaging biobanks. Eur Radiol Exp 6, 29 (2022). https://doi.org/10.1186/s41747-022-00281-1<br><br>R1 - COMMENT 2<br>2. The authors discuss in general terms the clinical use cases and cancers that are of interest for the data collection efforts, and mention some of the variables, but the complete list of the variables collected is not provided. Given that based on the Fig.1 the number of these variables is not exceedingly large, it would be appropriate to provide the complete list as an attachment to the manuscript.<br><br>We conceived this manuscript from a technical perspective (as technical note) and we considered that the description of the variables would benefit from an in-depth analysis of the clinical rationale behind that selection. The medical partners of the consortium are working on a specific manuscript with a clinical scope where we will provide a more detailed description of the variables, values and their clinical relevance. However, for technical usage, a basic list of the variables and the ontological codes used can be obtained within the script available in the github page:<br>https://github.com/EGA-archive/EuCanImage-FHIR/tree/main.<br><br>R1 - COMMENT 3<br>3. It would be rather interesting and important to better understand the use cases for utilization of the data collected.<br>* What are the operations that are expected to be performed on that data?<br><br>As suggested, for better understanding, we have included a more detailed description of the use cases (page 6 - Data description). The clinical data that is mentioned in this manuscript was collected as supporting information to complement the medical images, it contains the ground truth and serves as key supporting information to stratify patients into matched training or validation cohorts. It is, as well, additional data to be

Powered by Editorial Manager® and ProduXion Manager® from Aries Systems Corporation

	included as input in the AI models together with imaging data. The AI models try to respond the following questions:<br>-Hepatocellular carcinoma: Can AI increase the diagnostic sensitivity of liver MRI, currently at 60%, for detecting small HCC lesions (less than 20 mm) while keeping the specificity high?<br>-Colorectal liver metastasis: Can AI identify liver metastases in colorectal cancer from pre- and post-operative CT?<br>-Rectal cancer: Can AI predict the level of response to neoadjuvant radio(chemo)therapy based on primary MRI in rectal cancer for local staging and restaging?<br>-Rectal cancer - Lymph nodes metastasis: Can AI identify mesorectal lymph node metastases in pelvic MRI?<br>-Breast MRI: Could AI tools enable to de-escalate neoadjuvant systemic therapy (NST) in patients highly likely to achieve a pathological complete response (pCR)?<br>-Breast MMG: Can AI improve the assessment of screening mammograms by automatically differentiating benign from malignant lesions?<br>-Breast MMG - Molecular subtyping: Can AI distinguish five molecular subtypes of invasive ductal breast carcinoma on MMG?<br><br>*Who is the target user for this dataset?<br><br>The target users for this datasets are first, AI developers within EuCanImage, and then other AI researchers who would be interested in re-using this curated data, once the data is available in the permanent repository and after data access approval.<br><br>*What are the key capabilities that become possible due to adoption of FHIR for data representation? This would be appropriate in order to justify the selection of FHIR for the representation of the harmonization result.<br><br>The adoption of FHIR enables syntactic and semantic interoperability, making the confluence with other EU initiatives easier and thus providing more scalability. At the beginning of the project we evaluated some potentially suitable data standards OMOP, ICGC-ARGO and FHIR. In EuCanImage, the data modeling needed to serve two main purposes: a) harmonization and standardization of the data for unambiguous meaning and b) secondary use by being available under request in a repository, so, being in a format adequate for distribution. FHIR outperformed the other standards that were evaluated for the representation of concepts for this clinical domain and presented significant advantages for sustainable storage and data sharing at the permanent repository, EGA. The rationale has been included in page 9 - Data Model Design and in response to reviewer 2 comment 15.<br><br>*Examples/use cases of what is possible now that was not possible before would be beneficial.<br><br>This workflow (from raw data to the storage of standardized data at EGA) provides a new approach to integrate and share data using different already existing components from for data capture (REDCap) to data standardization (FHIR) and storage in an existing permanent repository (EGA), together with new developed scripts to fill the gaps (ETL script), which, all together, favors the sustainability of the project and data FAIRness. Without this workflow we couldn’t have used the data from the different hospitals due to non-compatible data format and unstandardized terminology.<br><br>R1- COMMENT 4<br>4. Related to point 2 above, since the authors developed the dictionary and the value sets for the variables being harmonized, it would be highly relevant to include those dictionaries and value sets as an attachment/appendix to the manuscript.<br><br>See response to comment 2<br><br>R1 - COMMENT 5<br>5. It appears that the effort of mapping the site-specific variables is primarily done at the time data is collected into CSV or is submitted using the online forms. It is not entirely clear what is the effort for such harmonization, what is the logistics of this harmonization (ie, is this performed entirely by the site personnel or there is a central

helpdesk that consults submitters on any questions arising). Further, given that the sites are located in different countries with different main languages, it would be helpful if authors could comment on the language barrier or the absence of such while collecting, harmonizing and submitting the data.

Thank you for pinpointing this, we have included additional insights to better describe the process in the revised manuscript (page 11 - Technical implementation of data standards and Figure 4 legend). Briefly, the harmonization efforts vary a lot between centers and use cases. Some sites had structured repositories with variables linked to standard terminologies that required minimal mapping and transformation efforts, while others performed this task manually or through dictionaries that mapped local concepts to standard ones. In all cases, this was performed by site personnel, but with guidance from the Data Model Working Group of EuCanImage. Regarding the language barrier, many of the concepts from the terminologies that were used have multilingual descriptions, which reduced language barriers. In the rest of the cases, mappings were performed by personnel who understood the meaning of standard terms in English and their local equivalence.

R1 - COMMENT 6
6. Since the overall focus of the EuCanImage is on radiological data, and since presumably such data would be longitudinal, it is important to provide details on how de-identification related activities are coordinated with the collection of clinical data, and how key attributes of the data (ie, various identifiers and dates) are kept in sync between different data types.

We described the key de-identification methods we specifically used for the clinical data, basically a) the use of EuCanImage ID to codify patient IDs (page 12) and 2) the de-identification of dates by using time periods or the use of arbitrary dates to mimic the Epoch Unix system (page 11). The imaging data required for the use cases is not longitudinal, for each patient we have only one set of images collected at one time point. The specific methods for the de-identification of medical images is out of the scope of this manuscript, however, imaging data de-identification tools and methods were described in detail and compared with other AI4HI network projects in a separate image de-identification manuscript currently under review.

R1 - COMMENT 7
7. While it provides interesting information, the section "Legal interoperability" seems to be somewhat disconnected from the rest of the narrative.

We agree with the reviewer's assessment. Although this section about the legal aspects is not directly related with the main focus of the manuscript (data standardization and interoperability challenges) we considered it to be of interest for the reader. In EuCanImage, ethico-legal discussions have played an important role from the beginning of the project and continue to be a recurring topic in similar consortia and initiatives. In addition to the GDPR, the European legal framework is increasingly populated with regulations and norms that directly relate to the scope of this paper. With this section our aim is to provide an overview on legal and practical issues that we have identified during the project (e.g., a rapidly evolving regulatory environment, the anonymisation-pseudonymisation conundrum in the GDPR, legal barriers on secondary uses of personal data for AI research and development, cross-border data sharing etc.) and that are relevant when trying to implement data standards for data interoperability.

In response to the reviewer's prescient feedback and for better integration and contextualization we have replaced the headers which could be misleading and included an introductory paragraph to better frame the purpose of this section and its coherence with the rest of the manuscript (pages 13-14).

Reviewer #2: This work can be of interest for researcher planning to develop similar data infrastructures.

From my point of view the main limitations of this work are:

R2- COMMENT 1

Powered by Editorial Manager® and ProduXion Manager® from Aries Systems Corporation

	* The lack of discussion around the OMOP CDM<br><br>We appreciate the remark and extended the discussion around the rationale behind standards selection. See response to comment 15, also page 9 of the manuscript.<br><br>R2- COMMENT 2<br>* The lack of overview and details about the ETL process<br><br>We provided a more detailed description of the ETL. For further insights into this process, first we need to specify that we can refer to two different types of ELT pipelines in the project 1) from hospital raw data to RedCap and 2) from the harmonized RedCap database to the standardized FHIR compliant file. The first ETL process was carried out directly by the clinical institutions with their own capabilities for doing an automatized data retrieval process. Description included in page 11. The second ETL process is common for all sites and it is performed centrally. For a more detailed description we added a specific section in methods (pages 18-19).<br><br>R2- COMMENT 3<br>* Limited reference to the FAIR data principles<br><br>We included a specific paragraph in the background (page 4). Interoperability is the focus of this work and we agree it was important to highlight the commitment of the project to the FAIR framework.<br><br>R2- COMMENT 4<br>* The claim that it is for precision oncology while it seems much more centered around imaging.<br><br>As the reviewer points out EuCanImage aims to build a platform around imaging data and the development of AI models in oncology. We appreciate the remark and for accuracy we have modified the wording, accordingly, throughout the document. See also comment 6.<br><br>Some more specific comments:<br><br>BACKGROUND<br>R2- COMMENT 5<br>* The reference 1 does not seem to back-up your claim that "Artificial intelligence (AI) for precision oncology is an exponentially growing field"<br><br>Thank you for pinpointing this out, we have updated the reference to:<br>Artificial Intelligence in Oncology: Current Landscape, Challenges, and Future Directions. William Lotter; Michael J. Hassett; Nikolaus Schult; Kenneth L. Kehl; Eliezer M. Van Allen ; Ethan Cerami. Cancer Discov (2024) 14 (5): 711–726. (https://doi.org/10.1158/2159-8290.CD-23-1199)<br><br>R2 - COMMENT 6<br>* Are we talking about precision medicine or precision oncology? And it is not very clear what you mean with precision oncology, typically is genomics based oncology, you should probably make it more explicit what you mean.<br><br>We agree with the perspective of the reviewer and we think this is a valuable appreciation, in accordance, we have refined the wording throughout the manuscript to be more explicit and accurate.<br><br>R2 - COMMENT 7<br>* I don't see your point about the broad implementation, typically data sharing is not for implementation but for research so I'm a bit confused here.<br><br>Thank you for pointing this out, we have simplified the sentence for concision (page 4). With implementation we refer to the process of translating the conceptual model we first developed (list of variables, the values, the codes…) into its actual application which also implies moving data between borders. Being sure that we were capable of fulfilling all the steps from data capture at hospitals, to data standardization and

Powered by Editorial Manager® and ProduXion Manager® from Aries Systems Corporation

storage, testing that the concept was feasible using existing technologies when possible and creating custom scripts to cover the gaps when needed.

R2 - COMMENT 8
* "Generating and collecting the mentioned data types": all structured data can be stored as CSV, so the problem is to combine that with genomics (VCF?) and imaging (NIFTI?)

The text reads as follows: "Generating and collecting the mentioned data types involve a multitude of different technological platforms and their storage in a wide range of data formats and information systems, contributing to increased data diversity". With this sentence we wanted to highlight the vast diversity of clinical data that is collected at healthcare institutions, which not only includes different data types but also the different technological platforms that are used in the different institutions (ie: different vendors, versions…). All that diversity increases the complexity of the harmonization and standardization requirements. For clarity we have rephased specifying it refers to the diversity of technological platforms and storage systems among hospitals (data sources) not in other contexts.

R2 - COMMENT 9
* "cross-border legal constraints and massive data volumes required", I don't think this is related to interoperability, you should probably rephrase that part.

We see the point of the reviewer. Thank you for pinpointing this out. In accordance, we have deleted the sentence for clarity and concision (page 4).

R2 - COMMENT 10
* "such as the Health Level Seven (HL7®) or fast healthcare interoperability resources (FHIR®) specification", HL7 develops the FHIR standard, you should reprhase that.

Thank you for pinpointing this inaccuracy. We have corrected the sentence (page 4-5).

DATA DESCRIPTION
R2 - COMMENT 11
* You mention the 7 key unmet needs and I'm a bit struggling to find them, you should make it explicit where to find them.

We agree with the reviewer, we have specified the aim of the different use cases in the data description section (page 6). For additional details, the aims are also described in response to reviewer 1 comment 3.

R2 - COMMENT 12
* "The dialogue between physicians and AI developers on clinically relevant variables that can be meaningfully incorporated in AI algorithms, with the GDPR-compliant data minimisation principle, led to the final set of defined variables (Figure 1)." You should detail more this process, was it a consensus from 100 clinicians across Europe or 2 clinicians from the main center giving their opinion? Also the actual list would be of interest, you should put that in supplementary.

We appreciate the suggestion and we have included that information in page 7. Briefly, at the beginning of the EuCanImage project it was created a Clinical Working group encompassing clinical representatives from five clinical centers. Each clinical site delegated their specialists towards each use case. Specialists means radiologist, pathologist, clinical oncologist, surgical oncologist, radiotherapy specialist and the data managers. In general the Clinical Working Group meetings were attended by 10-20 doctors every two weeks for the first year of the project. After the general concepts of use cases were established, we separated breast, colorectal and liver subgroups of the Clinical Working Group into organ-related meetings with different specialists per use case and center. These organ-related specialized groups met every two weeks for the next six months to develop the final list of clinico-pathological variables that were deemed to provide ground truth and provide clinical data adding value to the imaging data in deep learning modeling.
Regarding the final list of clinical variables, we considered that it would be essential to provide an adequate clinical context for the selection of the variables and delve into its

clinical relevance. The medical partners of the consortium are working on a specific manuscript with a clinical scope containing the precise variables, therefore we would prefer not to disclose this final information at the moment if that's acceptable. However, for a technical perspective, a basic list of the variables and ontological codes used can be obtained in the script available on the GitHub page: https://github.com/EGA-archive/EuCanImage-FHIR/tree/main.

R2 - COMMENT 13
* "between 8 and 39 parameters per use case", that sounds quite few for AI applications.

We understand the reviewer's point and have rephrased the text for clarification (page 7). The range between 8 and 39 parameters per use case only refers to the clinical attributes (what we call non-imaging data). Those variables, among other key information, contain the ground truth and will be integrated with more parameters extracted from medical images (MRI, CT, or MMG) and DICOM metadata to generate the AI models.

R2 - COMMENT 14
* "Next, the value sets for each clinical variable were discussed in detail and defined to ensure understanding and a manageable range of values" what does it mean? That you constraine the values to a specific range where you cap the outliers? I feel the process could be better described.

Thank you for the suggestion. We have rephrased the sentence to include a more specific description of the process (page 7). Briefly, the values of certain variables were categorized into value sets in order to be able to standardize their representation and link such concepts with a terminological code.

DATA CURATION
Analysis of semantic interoperability and health standards
R2 - COMMENT 15
* You gloss over the OMOP CDM part while I believe this is a key point, why did not you chose to build upon that instead?

We carefully considered OMOP CDM as a potential standard to represent EuCanImage data. Both OMOP and FHIR allow the structuring and formatting of healthcare data, however they serve fairly different purposes. While OMOP is more oriented towards the representation (structure and content) of clinical data, FHIR is more focused on healthcare data exchange. In EuCanImage, the data modeling needed to serve two main purposes: a) harmonization and standardization of the data for unambiguous meaning and b) secondary use by being available under request in a repository, so, being in a format adequate for distribution. The decision of choosing FHIR over OMOP was not obvious from the beginning, one of the aspects we considered to make the decision was the data sharing potential. While OMOP presented good performance for data representation it supposed an additional challenge to meet our second aim, data sharing. One of the interesting points of EuCanImage is that the platform is built over existing repositories, favoring in a great measure its sustainability. For the re-use of clinical data EGA is the permanent repository where it will be stored, in this regard FHIR presented significant advantages for sustainable storage and sharing once the project ends. Additionally, the existence of FHIR to OMOP (and OMOP to FHIR) conversion could solve the interoperability issue between the two main schemas.

Data Model Design
R2 - COMMENT 16
* "we decided to use FHIR due to its wide adoption, flexibility and suitability for real-world data exchange." This is why I wouldn't call FHIR a CDM, it is too flexible.

In the previous point we elaborate the basis of our decision to use FHIR, the rationale is also included in page 9 of the manuscript.

	R2 - COMMENT 17<br>* "Clinical elements necessary for each hypothesis were established by domain experts and interdisciplinary teams including clinicians and AI developers, considering different key data aspects" you need to explain that in more details, which key data aspects?<br><br>Thank you for the suggestion, we have further explained the important aspects for the selection of clinical elements in page 9. Briefly:<br>-Sample size<br>-Target population selection and characterization<br>-Clinical endpoints: pathological hallmarks, disease behavior, treatment response, patient prognosis…<br>-Type of outcome: binary, continuous, time to event<br>-Adequate ground truth<br>-Minimal amount of data principle<br>-Availability of specific variables at the data sources - for example in Sweden some cancer data comes from the national registries not the hospital itself.<br><br>R2 - COMMENT 18<br>* "When choosing the most suitable resource for each selected variable, each resource's constraints were considered", you need to give an example<br><br>Thank you for the suggestion. We have included an example of a resource's selection based on related content and constraints when choosing a suitable FHIR Resource in page 9-10.<br><br>TECHNICAL IMPLEMENTATION OF DATA STANDARDS<br>R2 - COMMENT 19<br>* "To minimise the need to re-encode and simplify the data capture process, we created electronic case-report forms (eCRF) with REDCap." as you mention, this will require a lot for manual work that will be a big problem for scability and portability. It is a good tool for research but not that much for implementation as you are claiming the project is about.<br><br>We agree that data capture with eCRF forms is, in general, an intensive manual work and can be a problem for scalability. In this regard, there were differences in the capabilities of the different hospitals to extract data from EHR and to transfer into REDCap, while some centers used REDCap in a more classical manual way, others with more structured databases could automate the process with their own scripts. So, in some way, the extent of manual work was dependent on the capabilities of the different centers. In this workflow, REDCap was at the same time a tool for data capture and a common database used as a proxy for data harmonization. It is also important to specify that in this context, implementation refers to the translation of the plan (the conceptual model) to its execution, regardless of the scale.<br><br>DISCUSSION<br>R2 - COMMENT 20<br>* You claim to focus on interoperability but only mention the FAIR data principles at this stage. And there are already some litterature specifically about the FAIR data principles in precision oncology (for example DOI: 10.1093/bib/bbz044), it is probably a good idea to include that.<br><br>Thank you for the suggestion, we agree with the reviewer. Interoperability is a key part of the work and FAIR principles are a pillar of the project. We have included the suggested reference and spotlight the importance of the adherence to FAIR principles in the background (page 4).<br><br>R2 - COMMENT 21<br>* "The standardised datasets that we will make available" You need to explain when it will be available.<br><br>Data will be available under request approval at the end of the project. A sentence has been added for concretion.
**Additional Information:**	

Question	Response
Are you submitting this manuscript to a special series or article collection?	No
**Experimental design and statistics**<br><br>Full details of the experimental design and statistical methods used should be given in the Methods section, as detailed in our Minimum Standards Reporting Checklist. Information essential to interpreting the data presented should be made available in the figure legends.<br><br>Have you included all the information requested in your manuscript?	Yes
**Resources**<br><br>A description of all resources used, including antibodies, cell lines, animals and software tools, with enough information to allow them to be uniquely identified, should be included in the Methods section. Authors are strongly encouraged to cite Research Resource Identifiers (RRIDs) for antibodies, model organisms and tools, where possible.<br><br>Have you included the information requested as detailed in our Minimum Standards Reporting Checklist?	Yes
**Availability of data and materials**<br><br>All datasets and code on which the conclusions of the paper rely must be either included in your submission or deposited in publicly available repositories (where available and ethically appropriate), referencing such data using a unique identifier in the references and in the "Availability of Data and Materials" section of your manuscript.	Yes

Powered by Editorial Manager® and ProduXion Manager® from Aries Systems Corporation

Have you have met the above requirement as detailed in our Minimum Standards Reporting Checklist?	

Manuscript Click here to access/download;Manuscript;Manuscript_reviewed_20240724.

TITLE:

New implementation of data standards for AI in oncology. Experience from the EuCanImage project.

AUTHORS:

Teresa García-Lezana[1], Maciej Bobowicz[2], Santiago Frid[3], Michael Rutherford[4], Mikel Recuero[5], Katrine Riklund[6], Aldar Cabrelles[1], Marlena Rygusik[2], Lauren Fromont[1], Roberto Francischello[7], Emanuele Neri[7], Salvador Capella[8], Fred Prior[4], Jonathan Bona[4], Pilar Nicolas[5], Martijn P. A. Starmans[9], Karim Lekadir[10,11], Jordi Rambla[1,12] and EuCanImage Consortium.

AFFILIATIONS:

1. Centre for Genomic Regulation (CRG), The Barcelona Institute of Science and Technology, Dr.Aiguader 88, Barcelona 08003, Spain
2. 2nd Department of Radiology, Medical University of Gdansk, Mariana Smoluchowskiego 17, 80-214, Gdansk, Poland
3. Clinical Informatics Service, Hospital Clínic de Barcelona, Villarroel 170, 08036 Barcelona, Spain
4. Department of Biomedical Informatics, University of Arkansas for Medical Sciences, Little Rock, Arkansas, United States
5. Social and Legal Sciences Applied to the New Technosciences Research Group, University of the Basque Country (UPV/EHU), Bilbao, Spain
6. Department of DIagnostics and Intervention, Diagnostic Radiology, Umeå university, Umeå, Sweden
7. Academic Radiology, Department of Translational Research, University of Pisa, Via Roma 67, 56126 Pisa, Italy
8. Barcelona Supercomputing Center (BSC), Jordi Girona 29, 08034, Barcelona, Spain
9. Department of Radiology and Nuclear Medicine and Department of Pathology, Erasmus MC Medical Center, Rotterdam, the Netherlands
10. Artificial Intelligence in Medicine Lab (BCN-AIM), Departament de Matemàtiques i Informàtica, Universitat de Barcelona, Barcelona, Spain
11. Institució Catalana de Recerca i Estudis Avançats (ICREA), Passeig Lluís Companys 23, Barcelona, Spain.
12. Universitat Pompeu Fabra (UPF), Barcelona, Spain

E-MAIL ADDRESSES:

Teresa García-Lezana (corresponding author): teresa.garcia@crg.eu

Maciej Bobowicz: maciej.bobowicz@gumed.edu.pl

Santiago Frid: FRID@clinic.cat

Michael Rutherford: MWRutherford@uams.edu

Mikel Recuero: mikel.recuero@ehu.eus

Katrine Riklund: katrine.riklund@umu.se
Aldar Cabrelles: aldar.cabrelles@crg.eu
Marlena Rygusik: marlena.rygusik@gumed.edu.pl
Lauren Fromont: lauren.fromont@crg.eu
Roberto Francischello: roberto.francischello@med.unipi.it
Emanuele Neri: emanuele.neri@unipi.it
Salvador Capella: salvador.capella@bsc.es
Fred Prior: FWPrior@uams.edu
Jonathan Bona: JPBona@uams.edu
Pilar Nicolas: mariapilar.nicolas@ehu.eus
Martijn P. A. Starmans: m.starmans@erasmusmc.nl
Karim Lekadir: karim.lekadir@ub.edu
Jordi Rambla: jordi.rambla@crg.eu

FUNDING: This project has received funding from the European Union's Horizon 2020 research and innovation programme under grant agreement No 952103.

KEYWORDS: interoperability, data model, oncology, artificial intelligence, FHIR,

ABSTRACT:

Background: An unprecedented amount of personal health data, with the potential to revolutionise precision medicine, is generated at healthcare institutions worldwide. The exploitation of such data using artificial intelligence relies on the ability to combine heterogeneous, multicentric, multimodal and multiparametric data, as well as thoughtful representation of knowledge and data availability. Despite these possibilities, significant methodological challenges and ethico-legal constraints still impede the real-world implementation of data models. **Technical details:** The EuCanImage is an international consortium aimed at developing AI algorithms for precision medicine in oncology and enabling secondary use of the data based on necessary ethical approvals. The use of well-defined clinical data standards to allow interoperability was a central element within the initiative. The consortium is focused on three different cancer types and addresses seven unmet clinical needs. We have conceived and implemented an innovative process to capture clinical data from hospitals, transform it into the newly developed EuCanImage data models and then store the standardised data in permanent repositories. This new workflow combines recognized softwares (REDCap for data capture), data standards (FHIR for data structuring) and an existing repository (EGA for permanent data storage and sharing), with newly developed custom tools for data transformation and quality control purposes (ETL pipeline, QC scripts) to complement the gaps. **Conclusion:** This article synthesises our experience and procedures for healthcare data interoperability, standardisation and reproducibility.

BACKGROUND

Artificial intelligence (AI) for oncology is an exponentially growing field[1] built over large amounts of patient-related data. The volume and depth of personal health data necessary to support precision medicine is unprecedented, and the integration and analysis of such heterogeneous data types require a thoughtfully structured representation of knowledge[2,3]. Besides, data needs to be shared across diverse institutions and even across multiple nations, increasing the complexity to data integration and flows.

FAIR principles (Findability, Accessibility, Interoperability and Reusability) are the international reference that defines the best practices for data sharing. 'Findability' entails the automatic discovery of datasets and services. 'Accessibility', makes reference to the retrieval of the data possibly including an approval process. 'Interoperability', refers to the use of standards for data integration. Finally, 'Reusability', implies an adequate description of the data (metadata) to optimise their use[4]. The adherence to the FAIR principles is imperative and its application in health science and oncology is crucial for the development of data sharing platforms[5].

Personal health data includes a wide range of different data types, among others, demographic characteristics, patient's symptoms, diagnoses, laboratory results, medications, imaging data and genomics. Generating and collecting the mentioned data types involves a multitude of different technological platforms (e.g. different vendors) and their storage in a wide range of data formats and information systems used by the different healthcare facilities, contributing to increased data diversity[6]. In fact, healthcare data has been shown to be more heterogeneous than other types of research data[7]. This high data complexity makes interoperability of healthcare information a significant hurdle in the development of AI models[8,9]. The barrier is even more prominent in oncological observational research since cancer diagnoses require a set of attributes usually registered separately (imaging, histology, topology, grade, stage, and biomarkers), and the complex patient trajectory involves personalised treatment regimens[9].

Data needs to be harmonised at three levels to address the interoperability challenge: technical, syntactic and semantic. On one hand, requirements in technical interoperability facilitate basic data exchange conventions (file formats), and on the other hand, syntactic and semantic interoperability define data structure and the use of ontologies for unambiguous representation of medical concepts, respectively[10]. Across the different healthcare ecosystems, equivalent information can be represented in diverse ways. The use of standards that can be universally interpreted, both human and machine-readable, facilitates harmonisation efforts. The structured exchange of health-related data is supported by international standards, such as the Fast Healthcare Interoperability Resources (FHIR) specification developed by Health Level Seven (HL7®) international. FHIR defines the structure of medical data in modular components called "Resources"[11]. It is envisioned that

the FHIR framework can become critical for implementing AI technologies in the health sector, just as Digital Imaging and Communication in Medicine (DICOM) or Picture Archiving and Communication System (PACS) for imaging data[6].

The EuCanImage is a European Council Research and Innovation Action funded research project that comprises multidisciplinary teams with the overall aim of building a data sharing platform to be filled with over 20000 cases on cancer, and AI models integrating imaging, clinical and phenotypic data from five different EU countries to improve cancer patients' outcomes. Briefly, the EuCanImage platform integrates established data infrastructures: Collective Minds Radiology (CMRAD) platform for collaborative image annotation, the Eurobioimaging as image repository, the European Genome-phenome Archive (EGA) as repository for clinical and phenotypic data and the Open EBench platform for AI algorithms benchmarking. Here, we synthesise our experiences as an overview of the methods and challenges we identified while working towards the standardisation and interoperability of health data (clinical data) and implementing a data model for AI in large-scale oncology research.

DATA DESCRIPTION

Purpose for data collection and data description

EuCanImage is a complex project centred around addressing seven key unmet clinical needs in cancer imaging. A multidisciplinary team of different speciality physicians (radiologists, oncologists, radiotherapists, surgical oncologists, pathologists), sociologists, psychologists, AI developers, data scientists, Small and Medium Enterprises (SMEs), imaging and oncology research associations, as well as patients' organisations, collaborated to identify and refine core research questions. The consortium pinpointed the most urgent topics in liver, colon and breast cancer and designed seven clinical use cases to respond to each specific clinical need. More specifically, we concentrated our efforts on one use case on hepatocellular carcinoma addressing the detection of indeterminate small lesions, three on colorectal cancer: one aimed to identify liver metastasis from pre and post-operative CT in colorectal cancer patients, and two on rectal cancer to a) identify lymph node metastasis in contrast-enhanced rectal MRI and b) predict the response to neoadjuvant treatment and three on breast cancer to a) identify patients likely to achieve pathological complete response to de-escalate neoadjuvant systemic therapy based on the single point, pre-treatment contrast-enhanced MRI, b) automatically differentiate benign and malignant lesions in screening mammograms and c) distinguish molecular subtypes of breast cancer based on digital mammograms. Our ambition is to integrate clinical, pathological and genetic data (non-imaging data) and radiological images (imaging data) to build algorithms going beyond standard practice, allowing personalised approaches informed by the best quality data. The initial effort to obtain such high-quality data was dedicated to defining clinical consensus and requirements for the use cases with specifications of clinical data variables.

The mentioned factors necessitate a comprehensive data model incorporating multifactorial inputs from multiple data sources. In EuCanImage, data is submitted from six university hospitals in Italy, Lithuania, Poland, Spain (two sites), and Sweden, national registries and two research institutions from the Netherlands. Each of the centres uses its own imaging infrastructure and PACS as well as electronic or paper health records that include demographic, clinical, pathological and phenotyping information recorded in Health Information Systems.

Regarding the clinical data defined for each use, some common variables exist for all use cases: patient ID, biological sex, age at diagnosis, diagnosis, and pathology (ICD-O-3 codes). On the other hand, there are use case-specific variables such as the hormone receptor status, HER2 mutational status, or Ki67 status for breast cancer, or information on specific chemotherapy agents with dosing regimens. The dialogue between physicians and AI developers on clinically relevant variables that can be meaningfully incorporated in AI

algorithms, with the GDPR-compliant data minimisation principle, led to the final set of defined variables (Figure 1).

The selection of the clinical variables was a complex and lengthy procedure starting at the beginning of the EuCanImage project with the setup of the Clinical Working Group that included clinical representatives from all participating clinical centres. Each clinical site delegated representation of specialists: radiologists, pathologists, clinical oncologists, surgical oncologists, radiotherapy specialists and the data managers. In general, the Clinical Working Group meetings were attended by 10-20 doctors every two weeks for the first year of the project. After general concepts of the use cases were established, the Clinical Working Group was divided into organ-related meetings (breast, colorectal and liver subgroups) integrating different specialists per use case and centre. These organ-related specialised groups met every two weeks for the next six months to develop the final list of clinico-pathological variables using the Delphi consensus methodology. The final variables are deemed to provide both, the ground truth and the clinical data with additional value for deep learning (DL) modelling.

The final number of clinical variables used as ground truth or additional input parameters for DL, varies between 8 and 39 variables per use case. This clinical information will be used along with the information extracted from radiological images to build next generation deep learning models combining both the clinical and the imaging information at once. Three levels of data provision were defined: minimal, mandatory and recommended. The minimal set contains essential information from the pathology assessment of specimens, e.g., cancer vs. other findings and the presence of complete pathological response vs. partial or no response. It allows the assembly of standard-level algorithms primarily using imaging information as input with pathology information as ground truth. The mandatory set contains important enriching information. These are all variables that should be included as input together with cancer images for more advanced and complex algorithms. Finally, the recommended set addresses additional clinical data points (e.g., risk factors for breast cancer) and phenotyping information (PAM50 results) available only from selected centres but with adequate numbers of patients for AI research. This recommended set would create a very interesting and promising asset in the project repository for future research that is otherwise not readily available from other data repositories.

Next, for each clinical variable we defined comprehensive and detailed value sets to standardise concept representation and link the terms with ontological codes ensuring unequivocal understanding. It allows good description of cohorts and, at the same time, prevents very fine-grained stratification of data with limited instances and unbalanced distribution in some of the cohorts. This consensus approach represents a compromise between the need for a precise representation of the clinical range of disease presentations and the goals of data clarity and homogeneity.

DATA CURATION

Analysis of semantic interoperability and health standards

Semantic interoperability represents a remarkable challenge for medical research. Data captured through health information systems are usually stored in locally-modelled clinical repositories, mostly in non-structured ways, thus hindering cross-national data source integration and translational research. Health information standards play a crucial role in defining the structure and meaning of clinical information for it to be unequivocally interpreted by different systems. However, there is no single standard that solves every need in the biomedical field, but rather different standards that either complement each other or compete with one another. This includes standardised vocabularies and classifications and also health information standards. Examples of vocabularies include the Systematized Nomenclature of Medicine – Clinical Terms (SNOMED CT)[12] or International Classification of Diseases (ICD)[13], Logical Observation Identifiers Names and Codes (LOINC)[14], OHDSI Standardised Vocabularies, and International Cancer Genome Consortium[15] - Accelerating Research in Genomic Oncology (ICGC ARGO[16]). Examples of health information standards include HL7 FHIR[17] and open Electronic Health Records (openEHR)[18].

Vocabularies and classifications represent concepts that pertain to the biomedical domain in a standard fashion[19], although they require a common structure that provides the syntactic interoperability required to achieve semantic interoperability. Common Data Models (CDMs) serve as representations of collected data aimed at facilitating the exchange, pooling, sharing, or storing data from multiple sources, and can provide this common structure[20]. Health information standards also provide a syntactic base to allow the formal representation of the structure of clinical information and its meaning.

FHIR was introduced in 2011 by the standard-developing organisation HL7[17]. The information within FHIR is organised in basic building blocks named Resources. Those blocks define the structure of the contained information. Although it is widely used in health informatics, its uptake in research environments is less prevalent[21]. Most studies using FHIR in health research focus on clinical research (including clinical trials), and just about 12% are oncology-related[22]. In these studies, FHIR has been mainly used for standardisation and data capture and to a lesser extent for data analysis[22].

Observational Medical Outcomes Partnership (OMOP) enables the systematic analysis of disparate observational databases through a common data model and a closed dictionary of terminologies, vocabularies, and coding schemes. Several authors consider it an adequate data model for sharing data in electronic health record (EHR)-based longitudinal studies[20,23].

ICGC ARGO[16] is an initiative that provides a fixed schema for creating 15 clinical tables oriented to genomic oncology research, thus oriented for addressing cancer-specific issues in the representation of clinical data. Unlike other CDMs and health information standards, its use of standardised vocabularies and classifications is limited.

Data Model Design

Data standardisation within the project is required to a) support content organisation and subsequent development of AI algorithms, b) facilitate interoperability and c) the secondary use of the data (ie: data distribution under request in a repository). Both OMOP and FHIR are widely adopted standards in clinical settings, however they were conceived to serve different purposes. OMOP is more oriented towards clinical data representation (structure and content) and FHIR more centred on healthcare data exchange. After thoroughly evaluating various CDM alternatives, we decided to use FHIR due to its wide adoption, flexibility and suitability for real-world data exchange. More importantly, and the key aspect we considered to select FHIR over OMOP, was its appropriateness for permanent data storage and long-term data sharing through the repository.

As previously outlined, clinical elements necessary for each hypothesis were established by domain experts and interdisciplinary teams including clinicians and AI developers, considering different key data aspects. Some key considerations for variable selection were characterization of the target population, clinical endpoints (pathological hallmarks, disease behaviour, treatment response and the patient prognosis), type of outcome (binary, continuous, time to event), adequate ground truth, minimal amount of data principle and finally availability of specific variables at data sources. For the purpose of the project, data to cover the seven use cases was arranged following five different data schemas. As a general overview, the highest level components of the FHIR model are the Resources, which contain hierarchical sub-layers of descriptive elements for more detailed data classification. The content and format of a Resource has controlled properties, meaning that the different data elements and data types allowed need to follow specific requirements. To design the data architecture needed for each EuCanImage use case, the following FHIR resources were identified as relevant: Patient, Condition, Observation, Procedure, Medication Administration and Diagnostic report (Figure 2A). When choosing the most suitable resource for each selected variable, each resource's constraints were considered. For example, the classification of patients into case (cancer) or control (benign lesion) groups could be interpreted as part of the Condition Resource, information about a diagnosis, or Observation Resource, capturing results of the tests (mammogram). In this example, we considered it within the Condition Resource, despite including also the benign cases, so we could associate it with age at diagnosis. Once the variables were assigned to a Resource, they were mapped

to the appropriate FHIR element. In cases where the clinical variables could be assigned to more than one suitable profile (ex: Histological type), simplicity criteria were applied to minimise the number of Resources used. Many data elements within the FHIR Resources require coded values. Some are fixed values defined by the FHIR specification, but others require external ontologies. As a general rule, HL7/FHIR terminology was used in a few established fields, basically status profiles. SNOMED was the preferred terminology for general clinical concepts, ICD-O3 for histology, LOINC for some test observations and RxNORM for medication. We used NCIT when the concept did not exist in previous ontologies (Figure 2B). The summary of the different stages we followed to conceptualise the data model is described in Figure 3.

It is essential to point out that the project presented some particular needs not fully represented by FHIR (and standards ontologies), requiring alternative paths solutions to overcome limitations. The gaps identified relate to the fact that FHIR was designed to support interoperability and data exchange in healthcare rather than specifically focusing on research needs. The primary limitations we faced were a) the need to represent concepts without available standard terminology, b) variables not structured as in healthcare practice, c) the representation of dates to comply with the de-identification of personal data, d) the representation of not provided (missing) information, e) the implementation of the model without a FHIR server.

For each clinical variable, we defined the limited set of permissible values (value set) that this variable can adopt. Some of these value sets need to include ambiguous concepts for simplicity and data harmonisation reasons. An example is the term ‘other’, which is required to group less frequent or more irrelevant values. Those terms, isolated from additional context, suppose a challenge for interoperability. We used SNOMED post-coordinated expressions to build more specific clinical ideas by combining relevant terms with compositional grammar. Another challenge posed were concepts that are not used in healthcare but are essential to contextualise the specific use cases for research purposes and which are not captured by standard terminologies. Some examples are the variable ‘breast cancer subtype-by proxy’ to group breast cancer patients according to hormone receptor, Ki67 and HER2 expression levels or ‘time interval between the end of the neoadjuvant treatment and surgery’. Additional difficulties include tumour grading systems such as modified Ryan Scheme for Tumor Regression Grade, Miller and Payne's Tumor Regression Grade, Residual Cancer Burden class or grading of DCIS. Our approach was using the specific grading scales in NCIT, if available, or using generic grading scales in SNOMED (ex: grade 1 on a scale of 1 to 5), despite the fact it could affect interoperability.

There were some variables characterised under the Medication Administration Resource that presented significant difficulties to be represented as needed for the purpose of the project.

To detail the chemotherapy dosage, we required the total number of chemotherapy cycles, dose (amount of medication per dose) and the accumulation dose within the same ‘Medication administration’ entry. However, that Resource is designed to collect that information differently (single entry).

In compliance with GDPR, personal data pseudonymization entails the removal of indirect data identifiers, such as dates. The collection of exact dates was replaced by the collection time intervals (months, weeks, etc.). Most FHIR Resources allow time periods as valid data types, however the Resource: Medication Administration, only allows dates (*ddmmyy*). To fulfil this FHIR restriction we recodified the time periods into arbitrary dates starting on January 1st of 1970 to mimic the Epoch Unix system, with the end date calculated based on the collected time interval and starting date in mind.

TECHNICAL IMPLEMENTATION OF DATA STANDARDS

Transforming and loading ‘raw’ data from various hospital data systems into the newly developed data schema proved to be challenging and labour-intensive. The harmonisation efforts required by the different participating institutions and different use cases varied a lot depending on the existing resources at the sites. While some centres had structured repositories with variables linked to standard terminologies that required minimal mapping and transformation efforts, others performed the task of mapping local concepts with the standard ones manually. This effort was performed by trained site personnel understanding the meaning of the concepts in both English and local language. There was also an online support provided by the EGA when needed.

The FHIR implementation format has a hierarchical architecture, that, while having many advantages to encode the relationships between the variables and facilitating data storage, supposes an additional barrier for data providers given that most of the required information was not structured data inside their health records. To minimise the need to re-encode and simplify the data capture process, we created electronic case-report forms (eCRF) with REDCap. REDCap is a secure web application that supports data capture primarily for research studies[24,25]. This software allows the custom design of data entry forms and data collection workflows. It features a user-friendly interface to design the forms, field validation, custom logic patterns, calculated fields, data import/export options, data quality control and role-based user access. Additionally, it offers a set of APIs for integration with other platforms[26]. REDCap was deployed at the European Genome-phenome Archive (EGA) servers to design and manage data entry forms for clinical data collection within the consortium. Different data entry forms were conceived to support each of the five different data schemas.

Data from hospitals can be imported to the EuCanImage REDCap database following two paths: 1) by directly filling the online forms or 2) by entering data into CSV files complying with the specific REDCap format requirements, and then uploading the files into REDCap.
Patient IDs were previously pseudo-anonymised at the hospitals, and only hashed patient IDs (EuCanImage ID) were introduced in the platform. Consequently, all related clinical data from the different institutions merged into a single harmonised database for each use case. Once harmonised data was at the database quality control checks were performed. Then, all data was exported from REDCap as a CSV file for conversion into FHIR-compliant files.
To implement the FHIR model, we created each Resource using individual persistent identifiers with Uniform Resource Names (URN), more specifically with Universally Unique Identifiers (UUID). These identifiers were generated for each patient, resource and bundle. In FHIR, a bundle is a way to envelope all the Resources belonging to a single patient. In our case, a bundle is generated from a single row of the exported CSV files.
For the subsequent data standardisation stage, we built Extract Transform Load (ETL) pipelines to transform the output CSV files into JSON files compliant with the FHIR schema.
To automate this process, we used Python 3.11, and we followed a FHIR 4.3 schema. The python scripts are available on Github (https://github.com/EGA-archive/EuCanImage-FHIR/), with the additional use of external validators, such as FHIR Validator GUI (https://validator.fhir.org/) and Simplifier (https://simplifier.net/). A more detailed description of the steps followed for the creation of the ETL scripts is in methods. While building the Python scripts, the mapping of the dictionaries was hardcoded using FHIR-compliant ontologies. The results of the ETL process are JSON files containing the patients' information standardised to the CDM, one file per patient. Those files will be, on one side, the data source on which AI algorithm development relies, and on the other, standardised data available for the scientific community after proper data access request (Figure 4).

Data quality & consistency

Quality data can be defined as data that is fit for purpose, e.g., the data are sufficient for the specified purpose for which it is intended[27,28]. In most cases, data quality for the purpose of machine learning cannot be limited to a single focus but must cater to the needs of multiple audiences. Data quality issues can be introduced at any point in the data management and collection lifecycle. Whether during data acquisition, storage, analysis, or publication, diminished quality can inadvertently affect downstream tasks such as AI training[27–29].
We employ both quality assurance and quality control techniques over the course of the data life cycle including strict conformance to requirements during input and the assessment methods for repeatable feedback and improvement. The overall objective of our quality analysis is to rate the individual records based on multiple dimensions of quality and use this

as a filter for downstream tasks. To achieve the measure of data quality needed for superior AI training and results, we defined quality control rules and procedures based on standard dimensions of quality and built tools to integrate into our pipelines for data collection and storage.

Since we use REDCap as the intermediary data store where all collection methods funnel their data, we found REDCap's data quality module[24,25] valuable for organising our data quality rules. This module allows for the execution of quality checks for all data entered into the system, whether by direct entry or by CSV imports. This also enabled the capability to export these rules for use in customised tools for data collection.

Data quality can be evaluated over many different dimensions[28] and we have focused our evaluation on three critical dimensions: completeness, conformance and plausibility. For completeness, we focused on value requirements. For conformance, we analysed the various data types and permissible values to ensure adherence. Plausibility applied to ranges, such as age. The types and dimensions are outlined in Table 1.

Much of the needed data quality assessment functionality was already built into the REDCap quality module including pre-established rules handling blank values, data type errors, outliers and invalid permissible values. We also included custom rules covering multiple levels of requirements: minimal, mandatory and recommended. These rules aligned with the required fields outlined for each use case and agreed by representatives from each clinical centre.

After assessment of the data based on these organising quality criteria, we generate a score for each quality check based on the number of successes and failures. Here we can also apply weights if we deem an assessment more important than others. Table 2 shows an example of a scoring report.

LEGAL ASPECTS

In EuCanImage, ethico-legal discussions have played an important role from the beginning of the project and continue to be a recurring topic in similar consortia and initiatives. In addition to the well-known General Data Protection Regulation (GDPR), the last decade has witnessed the flourishing of a plethora of regulations and norms that are directly or indirectly relevant when trying to implement data standards for data interoperability. These include, among others, the recently passed Data Governance Act (DGA) and the Artificial Intelligence Act (AIA) and the proposal for a Regulation on the European Health Data Space (EHDS). Such regulatory developments are likely to be of considerable practical relevance to the scope of this paper, in particular, for discussions on the personal or non-personal nature of datasets involved, legal barriers on secondary uses of data for AI research and development and cross-border data sharing for AI research in oncology.

Personal data vs non-personal data.

As anticipated, early discussions within research projects and consortia often concern the personal or non-personal nature of the data to be processed. This is mainly due to the fact that processing operations involving personal data, namely information relating to an identified or identifiable natural person, fall under the scope of the GDPR. Therefore, non-personal data such as anonymised data (i.e., personal data rendered anonymous in such a manner that the data subject is not or no longer identifiable) are not bound by the Regulation. Pseudonymised data, which could be attributed to a natural person by the use of additional information, would qualify as personal data (see Article 4(5) and Recital 26 of the GDPR[30]). Generally, de-identification, which is understood as the process of removing or substituting all personal information and identifiers, is not, per se, sufficient to achieve the anonymisation threshold required in the EU. As a result, if raw data is retained at source or any key or additional information is reasonably likely to be used to reverse the process and re-identify the data subject, information shall be considered as pseudonymised data and thus subject to the GDPR.

Within EuCanImage, the removal of direct and indirect personal identifiers has been carried out. In addition, the data is double-hashed both by the data providers and by the platform.. Additionally, processing operations carried out within the project are actually aligned with the GDPR and supported by a contractual and governance structure that further enables data sharing.

Secondary use of data for AI research in precision medicine

Further processing of personal data for scientific research purposes, which comprises AI research in precision medicine as pursued by EuCanImage, is compatible with the GDPR (art. 5(1)(b)). Moreover, under forthcoming EHDS Regulation, secondary use of pseudonymised electronic health data is expressly permitted for "training, testing and evaluating of algorithms, including in medical devices, in vitro diagnostic medical devices, AI systems and digital health applications (art. 34(1)(e)). It should be noted, nonetheless, that these two terms ("further processing" and "secondary use") are not legally analogous, but the latter will be preferred here for the sake of clarity.

Cross-border data sharing for AI research in precision medicine

Despite the advent of the GDPR and the harmonisation effort, processing operations involving organisations and researchers from several EU Member states still face slight differences between national, regional or sectoral regulatory frameworks. Hence, although data sharing within Member states of the European Economic Area (EEA) is not hindered by any additional requirements, EuCanImage's partners and their legal teams still must cope with a complex

and fragmented scenario. Not only from a legal interoperability perspective, but also concerning divergent ethical oversight layers and internal procedures of each centre, hospital, institution or country.

Transfers of personal data to third countries or international organisations (i.e., data sharing with researchers or organisations located outside the EEA) remain a controversial issue[31,32]. Notwithstanding the fact that EuCanImage members do not plan to store or process data outside the EEA, controversies have arisen in relation to the transfer of data to international organisations and to UK-based institutions after Brexit. Potential routes for transfers remain limited, particularly, in light of the strict requirements and threshold set by the Court of Justice of the European Union[33,34].

DISCUSSION

In all scientific disciplines, but especially in health research, working with large scale datasets and engaging in cross-border data sharing is becoming increasingly vital for the adoption and development of AI technologies. EuCanImage focuses on leveraging existing healthcare data to address various scientific research questions using AI models. Our experience uncovers obstacles to data interoperability and reuse, as well as realistic solutions. In this report, we outline our procedures to achieve data interoperability, including a thorough description of the data model design, the standards used, harmonisation efforts, and methodological aspects concerning the practical implementation, along with legal interoperability considerations. The successful development and deployment of our data models and related standards represents a significant milestone, laying the groundwork for future AI applications in cancer healthcare. Furthermore, our work highlights the need for improvements in data collection, annotation, and cross-border dissemination.

We anticipate that our approach and methods will not only benefit individual institutions but also serve as a guide for future large-scale consortia requiring harmonisation and interoperability of cancer-related clinical data for AI and machine learning advancements. Similar efforts have been developed by other consortia[35], including projects within the AI4HI initiative such as Chaimilion, ProCancer-I, Incisive or Primarge[36]. These collective endeavours have all involved meticulous, collaborative, expert-driven analysis, spanning model design, data curation, standards usage, and infrastructure development. The knowledge and experience gained from our combined efforts are crucial in laying the groundwork for future healthcare data standardisation initiatives for AI research across Europe.

Achieving interoperability in healthcare data raises complex issues that need to be addressed. The development of supervised AI models trained for prediction or classification tasks relies on data labelled with ‘ground truth’ classifications. Reaching a consensus on data labelling requires common standard definitions for diagnosis and agreements on the level of data granularity; these are critical factors that affect the reproducibility and quality of the results[37,38]. Cancer diagnosis involves the integrating complex criteria based on a variety of disparate data components, such as pathology reports, laboratory results, radiology findings, and advanced molecular and genetic tests. Close collaboration among different medical specialists has enabled the establishment of key principles for data harmonisation: 1) the selection and definition of essential clinical variables to address the medical needs, 2) the identification of common data available across all centres and 3) striking a balance between the volume and granularity of the data that can be provided by various hospitals and the optimal information required for AI models (Figure 3).

Within the healthcare-research ecosystem, data sharing remains a barrier. Yet, it is a crucial mechanism for ensuring that high quality data, obtained through exhaustive and expensive processes like defining data labels, harmonisation tasks, and the use of common standards, can be reused by other researchers and thus, maximise the impact. The FAIR principles provide the framework for such data re-use[39]. Despite progress in adopting interoperability standards, data from different sources still contain discrepancies. To make data fully reusable and reproducible, methods for data cleaning, harmonisation, and standardisation must be transparent[40].

While the presented work demonstrates the feasibility of using HL7 FHIR to achieve interoperability, it also has limitations. FHIR resources were employed for structural interoperability, while SNOMED, LOINC, NCIT, and RxNorm were mainly used for semantic interoperability. By leveraging the comprehensive information model in FHIR, clinical data can be organised hierarchically in a manner that captures its context and remains unambiguous[41]. However, utilising FHIR for building a model for research oncology presents specific constraints and unique requirements for maintaining data interoperability (described previously - data model section). To maximise the potential of FHIR and encourage broader adoption in the specialised scientific context of AI for precision medicine, alternative FHIR configurations or detailed methodological explanations should be considered to ensure reproducibility. At this moment, the main limitation is the fact that the suitability of the models to develop the AI algorithms have not been validated yet.

In summary, we demonstrated that large-scale, real-world, multicenter clinical data harmonisation and curation for AI research is feasible through the use or adaptation of common standards. The standardised datasets that we will make available at the end of the project, which include data from over 20,000 cancer patients, will provide an invaluable resource for investigators. Expanding the understanding of these complex diseases and opening the door for cutting edge translational research beyond the scope of EuCanImage.

METHODS

Data model

The clinical data necessary to address each use case was established by interdisciplinary teams including clinicians and AI experts considering different key data aspects. Data was arranged following five different data schemas compliant with the FHIR (FHIR Release 4B) architecture. The FHIR Resources used were: Patient, Condition, Observation, Procedure, Medication Administration and Diagnostic report.

Ontologies

HL7/FHIR terminology was used in status profiles required by FHIR. SNOMED (SNOMED version International 2022-12-31) was the preferred terminology for general clinical concepts, ICD-O3 (ICD-O3 version 20220429) for histology, LOINC (LOINC version 2.73) for some test observations and RxNorm (RxNorm version 03-Jan-2023) for medication. We used NCIT (NCIT version 23.8d) when the concept did not exist in previous ontologies.

Data capture, standardisation and quality control

Patient IDs were pseudo-anonymised at the hospitals, and only hashed patient IDs (EuCanImage ID) were introduced in the platform. Data from hospitals was captured in REDCap [REDCap version 13.10.0; PHP 8.1.3 (Linux/Unix OS); MySQL 8.0.30] by filling the online forms or uploading CSV files complying with the specific format requirements. As a result, all clinical data merged into a single harmonised database for each data schema. At this stage we performed quality control checks. We focus our evaluation on three critical dimensions: completeness, conformance and plausibility and we generated a score for each quality check based on the number of successes and failures. We built ETL pipelines in python to transform the harmonised output data into JSON files compliant with FHIR. We used FHIR Validator GUI (https://validator.fhir.org/) and (https://simplifier.net/) as external validators for quality control. Code availability: The python scripts to transform harmonised data to the FHIR compatible schemas are available at https://github.com/EGA-archive/EuCanImage-FHIR/

Steps to create the ETL script

Step 0 - Dictionary: Before processing the data, we setted up an environment with the necessary resources. This includes the creation of a machine readable data dictionary encoding a) the name of the variable, b) the ontological code and c) the RedCap internal codification. This mapping served two purposes simultaneously 1) Data Quality Control and 2) the Extract Transform Load (ETL) process.

Step 1 - Parsing: All the data gathered in REDCap was exported into a CSV file per use case and clinical centre. These CSV files were parsed into a Python readable form for the posterior transformation to the objects that were required by the libraries.

Step 2 - Dictionary import: In order for the transformations to happen with minimal amount of errors, dictionaries from step 0 were imported into the Python script for the consecutive mapping to the FHIR Resources.

Step 3 - FHIR Resource mapping: To streamline the transformation of the different types of data variables into their respective FHIR Resources, we defined functions to automate the process.

1. First, empty templates were created for each FHIR Resource type. To avoid errors, we used maintained libraries that followed FHIR structures with internal validators .
2. Then, depending on the input required by each function (such as information about medication administrations, quantitative or qualitative observations, conditions from the patients or others), the Resource was populated accordingly. Additionally, some variables needed some extra processing, such as parsing dates and timestamps, also automated by the code.
3. In the case of an error in the structure, the libraries flag them for correction.

Step 4 - Export and validation: As a final step, the objects that were created in the script needed to be exported into JSON files. Given that the code included FHIR libraries streamlining the process, parsing the generated objects and dictionaries into the JSON file was straightforward. The libraries used in the process validate the integrity of the structure, but not always of the contents. To confirm the correctness of the result, we used external validators, such as FHIR Validator GUI (https://validator.fhir.org/) and Simplifier (https://simplifier.net/).

European Genome-Phenome Archive

The European Genome-phenome Archive (EGA) is a service for permanent archiving and sharing of personally identifiable genetic, phenotypic, and clinical data. The standardised clinical data, one JSON file (FHIR compliant) per patient, obtained after the previously described process will be stored encrypted at the EGA repository.

ACKNOWLEDGEMENTS

We acknowledge support of the Spanish Ministry of Science and Innovation through the Centro de Excelencia Severo Ochoa (CEX2020-001049-S, MCIN/AEI /10.13039/501100011033), and the Generalitat de Catalunya through the CERCA

programme. We are grateful to the CRG Core Technologies Programme for their support and assistance in this work.

We acknowledge the Social and Legal Sciences Applied to the New Technosciences Research Group, University of the Basque Country (UPV/EHU). Grant from the Department of Education of the Basque Government to support the activities of Research Groups from the Basque University System (Reference IT 1541-22)

This project has received funding from the European Union's Horizon 2020 research and innovation programme under grant agreement No 952103.

AUTHOR CONTRIBUTIONS

J.R., L.F. and T.G.L. developed the concept. F.P., K.L., M.S., M.B., and S.C. substantially contributed to the development of the concept. M.B., K.R., S.F., E.N. provided clinical advice. J.R., L.F., T.G.L., M.B., K.R., S.F. and R.F. conceptualised the data model. A.C. and M.Ry. Implemented the model and tests. A.C. and M.Ru. wrote the code. M.Ru., F.P., J.B. and A.C. conceptualised and implemented quality control. M.Re. and P.L. provide legal guidelines. T.G.L., M.B., S.F., M.Ru., M.Re. and A.C. conceptualised and wrote the manuscript. T.G.L. and M.B. created figures. All authors contributed to the critical revision of the manuscript.

COMPETING INTERESTS

The authors declare no competing interests

FIGURE LEGENDS

Figure 1. Data minimisation of clinical (non-imaging) parameters

Figure 2. A) Representation of the proportion of FHIR resources needed for each use-case **B)** Ontologies used in each resource

Figure 3. Description of the different steps followed to conceptualise the data model

Figure 4. Clinical data processing workflow from clinical institutions to data analysis/sharing. Hospitals import data to REDCap following different paths depending on local resources 1) some centres introduce data manually (filling online forms or CSV files) or 2) develop their own ETL scripts to automate the process. As a result all clinical data from the different institutions merge into a single harmonised database for each use case. Finally, all data is exported from REDCap as a CSV file for its standardisation and conversion into FHIR-compliant files (FHIR JSON) and stored at the European Genome-Phenome Archive (EGA).

REFERENCES

1. Lotter W, Hassett MJ, Schultz N, Kehl KL, Van Allen EM, Cerami E. Artificial Intelligence in Oncology: Current Landscape, Challenges, and Future Directions. *Cancer Discov*. 2024; doi: 10.1158/2159-8290.CD-23-1199.

2. Haendel MA, Chute CG, Robinson PN. Classification, Ontology, and Precision Medicine. *N Engl J Med*. 2018; doi: 10.1056/NEJMra1615014.

3. Dinov ID. Methodological challenges and analytic opportunities for modeling and interpreting Big Healthcare Data. *GigaScience*. 2016; doi: 10.1186/s13742-016-0117-6.

4. Wilkinson MD, Dumontier M, Aalbersberg IjJ, Appleton G, Axton M, Baak A, et al.. The FAIR Guiding Principles for scientific data management and stewardship. *Sci Data*. Nature Publishing Group; 2016; doi: 10.1038/sdata.2016.18.

5. Vesteghem C, Brøndum RF, Sønderkær M, Sommer M, Schmitz A, Bødker JS, et al.. Implementing the FAIR Data Principles in precision oncology: review of supporting initiatives. *Brief Bioinform*. 2020; doi: 10.1093/bib/bbz044.

6. He J, Baxter SL, Xu J, Xu J, Zhou X, Zhang K. The practical implementation of artificial intelligence technologies in medicine. *Nat Med*. 2019; doi: 10.1038/s41591-018-0307-0.

7. Kruse CS, Goswamy R, Raval YJ, Marawi S. Challenges and Opportunities of Big Data in Health Care: A Systematic Review. *JMIR Med Inform*. 2016; doi: 10.2196/medinform.5359.

8. Sweeney SM, Hamadeh HK, Abrams N, Adam SJ, Brenner S, Connors DE, et al.. Challenges to Using Big Data in Cancer. *Cancer Res*. 2023; doi: 10.1158/0008-5472.CAN-22-1274.

9. Frid S, Bracons Cucó G, Gil Rojas J, López-Rueda A, Pastor Duran X, Martínez-Sáez O, et al.. Evaluation of OMOP CDM, i2b2 and ICGC ARGO for supporting data harmonization in a breast cancer use case of a multicentric European AI project. *J Biomed Inform*. 2023; doi: 10.1016/j.jbi.2023.104505.

10. Näher A-F, Vorisek CN, Klopfenstein SAI, Lehne M, Thun S, Alsalamah S, et al.. Secondary data for global health digitalisation. *Lancet Digit Health*. 2023; doi: 10.1016/S2589-7500(22)00195-9.

11. Ayaz M, Pasha MF, Alzahrani MY, Budiarto R, Stiawan D. The Fast Health Interoperability Resources (FHIR) Standard: Systematic Literature Review of Implementations, Applications, Challenges and Opportunities. *JMIR Med Inform*. 2021; doi: 10.2196/21929.

12. SNOMED International. https://www.snomed.org Accessed 2024 Feb 1.

13. International Classification of Diseases (ICD). https://www.who.int/standards/classifications/classification-of-diseases Accessed 2024 Feb 1.

14. LOINC. LOINC. https://loinc.org/ Accessed 2024 Feb 5.

15. Reich C, Ostropolets A, Ryan P, Rijnbeek P, Schuemie M, Davydov A, et al.. OHDSI

Standardized Vocabularies-a large-scale centralized reference ontology for international data harmonization. *J Am Med Inform Assoc JAMIA*. 2024; doi: 10.1093/jamia/ocad247.

16. : ICGC ARGO. https://www.icgc-argo.org/ Accessed 2023 Oct 5.

17. FHIR. https://www.hl7.org/fhir/ Accessed 2023 Aug 30.

18. openEHR International. https://www.openehr.org/about/what_is_openehr Accessed 2024 Feb 1.

19. Plazzotta F, Luna D, González Bernaldo de Quirós F. [Health information systems: integrating clinical data in different scenarios and users]. *Rev Peru Med Exp Salud Publica*. 32:343–512015;

20. Garza M, Del Fiol G, Tenenbaum J, Walden A, Zozus MN. Evaluating common data models for use with a longitudinal community registry. *J Biomed Inform*. 2016; doi: 10.1016/j.jbi.2016.10.016.

21. Duda SN, Kennedy N, Conway D, Cheng AC, Nguyen V, Zayas-Cabán T, et al.. HL7 FHIR-based tools and initiatives to support clinical research: a scoping review. *J Am Med Inform Assoc JAMIA*. 2022; doi: 10.1093/jamia/ocac105.

22. Vorisek CN, Lehne M, Klopfenstein SAI, Mayer PJ, Bartschke A, Haese T, et al.. Fast Healthcare Interoperability Resources (FHIR) for Interoperability in Health Research: Systematic Review. *JMIR Med Inform*. 2022; doi: 10.2196/35724.

23. OMOP Common Data Model. https://ohdsi.github.io/CommonDataModel/index.html Accessed 2023 Oct 5.

24. Harris PA, Taylor R, Minor BL, Elliott V, Fernandez M, O'Neal L, et al.. The REDCap consortium: Building an international community of software platform partners. *J Biomed Inform*. 2019; doi: 10.1016/j.jbi.2019.103208.

25. Harris PA, Taylor R, Thielke R, Payne J, Gonzalez N, Conde JG. Research electronic data capture (REDCap)—A metadata-driven methodology and workflow process for providing translational research informatics support. *J Biomed Inform*. 2009; doi: 10.1016/j.jbi.2008.08.010.

26. Cheng AC, Duda SN, Taylor R, Delacqua F, Lewis AA, Bosler T, et al.. REDCap on FHIR: Clinical Data Interoperability Services. *J Biomed Inform*. 2021; doi: 10.1016/j.jbi.2021.103871.

27. Talburt JR. Entity Resolution and Information Quality. Elsevier;

28. Bian J, Lyu T, Loiacono A, Viramontes TM, Lipori G, Guo Y, et al.. Assessing the practice of data quality evaluation in a national clinical data research network through a systematic scoping review in the era of real-world data. *J Am Med Inform Assoc JAMIA*. 2020; doi: 10.1093/jamia/ocaa245.

29. Priestley M, O'donnell F, Simperl E. A Survey of Data Quality Requirements That Matter in ML Development Pipelines. *J Data Inf Qual*. 2023; doi: 10.1145/3592616.

30. Regulation (EU) 2016/679 of the European Parliament and of the Council of 27 April 2016

on the protection of natural persons with regard to the processing of personal data and on the free movement of such data, and repealing Directive 95/46/EC (General Data Protection Regulation). 2016 May 4;

31. Bernier A, Molnár-Gábor F, Knoppers BM, Borry P, Cesar PMDG, Devriendt T, et al.. Reconciling the biomedical data commons and the GDPR: three lessons from the EUCAN ELSI collaboratory. *Eur J Hum Genet*. Nature Publishing Group; 2024; doi: 10.1038/s41431-023-01403-y.

32. Molnár-Gábor F, Beauvais MJS, Bernier A, Jimenez MPN, Recuero M, Knoppers BM. Bridging the European Data Sharing Divide in Genomic Science. *J Med Internet Res*. 2022; doi: 10.2196/37236.

33. Court of Justice of the European Union, Judgement of 16 July 2020, Case Schrems II (C-311/18).

34. European Data Protection Board (EDPB), Recommendations 01/2020 on measures that supplement transfer tools to ensure compliance with the EU level of protection of personal data, adopted on 18 June 2021.

35. Sweeney SM, Hamadeh HK, Abrams N, Adam SJ, Brenner S, Connors DE, et al.. Case Studies for Overcoming Challenges in Using Big Data in Cancer. *Cancer Res*. 2023; doi: 10.1158/0008-5472.CAN-22-1277.

36. Kondylakis H, Ciarrocchi E, Cerda-Alberich L, Chouvarda I, Fromont LA, Garcia-Aznar JM, et al.. Position of the AI for Health Imaging (AI4HI) network on metadata models for imaging biobanks. *Eur Radiol Exp*. 2022; doi: 10.1186/s41747-022-00281-1.

37. Wang SY, Pershing S, Lee AY, Committee on behalf of the AT on A and AMIT. Big data requirements for artificial intelligence. *Curr Opin Ophthalmol*. 2020; doi: 10.1097/ICU.0000000000000676.

38. Cirillo D, Núñez-Carpintero I, Valencia A. Artificial intelligence in cancer research: learning at different levels of data granularity. *Mol Oncol*. 2021; doi: 10.1002/1878-0261.12920.

39. Scheffler M, Aeschlimann M, Albrecht M, Bereau T, Bungartz H-J, Felser C, et al.. FAIR data enabling new horizons for materials research. *Nature*. 2022; doi: 10.1038/s41586-022-04501-x.

40. Heacock ML, Lopez AR, Amolegbe SM, Carlin DJ, Henry HF, Trottier BA, et al.. Enhancing Data Integration, Interoperability, and Reuse to Address Complex and Emerging Environmental Health Problems. *Environ Sci Technol*. American Chemical Society; 2022; doi: 10.1021/acs.est.1c08383.

41. Leroux H, Metke-Jimenez A, Lawley MJ. Towards achieving semantic interoperability of clinical study data with FHIR. *J Biomed Semant*. 2017; doi: 10.1186/s13326-017-0148-7.

Click here to access/download;Table;Table 1.docx

Table 1. Clinical data quality assessment Dimensions

Type	Dimension	Description
Minimal_req	Completeness	Meets minimum requirements
Mandatory_req	Completeness	Meets mandatory requirements
Length	Conformance	Conforms to length restrictions
Datatype	Conformance	Conforms to data type restrictions
Permissible	Conformance	Conforms to list of permissible values
Range	Plausibility	Meets known range limits

Click here to access/download;Table;Table 2.docx

Table 2. Data quality scoring report

	Dimension	Type	Fail	Pass	Total	Weight	Score
0	Completeness	minimal_req	2	16	18	50	44.44%
1	Completeness	mandatory_req	3	32	35	10	9.14%
2	Conformance	length	1	10	11	10	9.09%
3	Conformance	datatype	0	40	40	10	10.00%
4	Conformance	permissible	3	52	55	10	9.45%
5	Plausibility	range	1	3	4	10	7.50%
**Total:**			**10**	**153**	**163**	**100**	**89.63%**

Click here to access/download;Figure;Figure 1_20240216.pdf

FIGURE 1

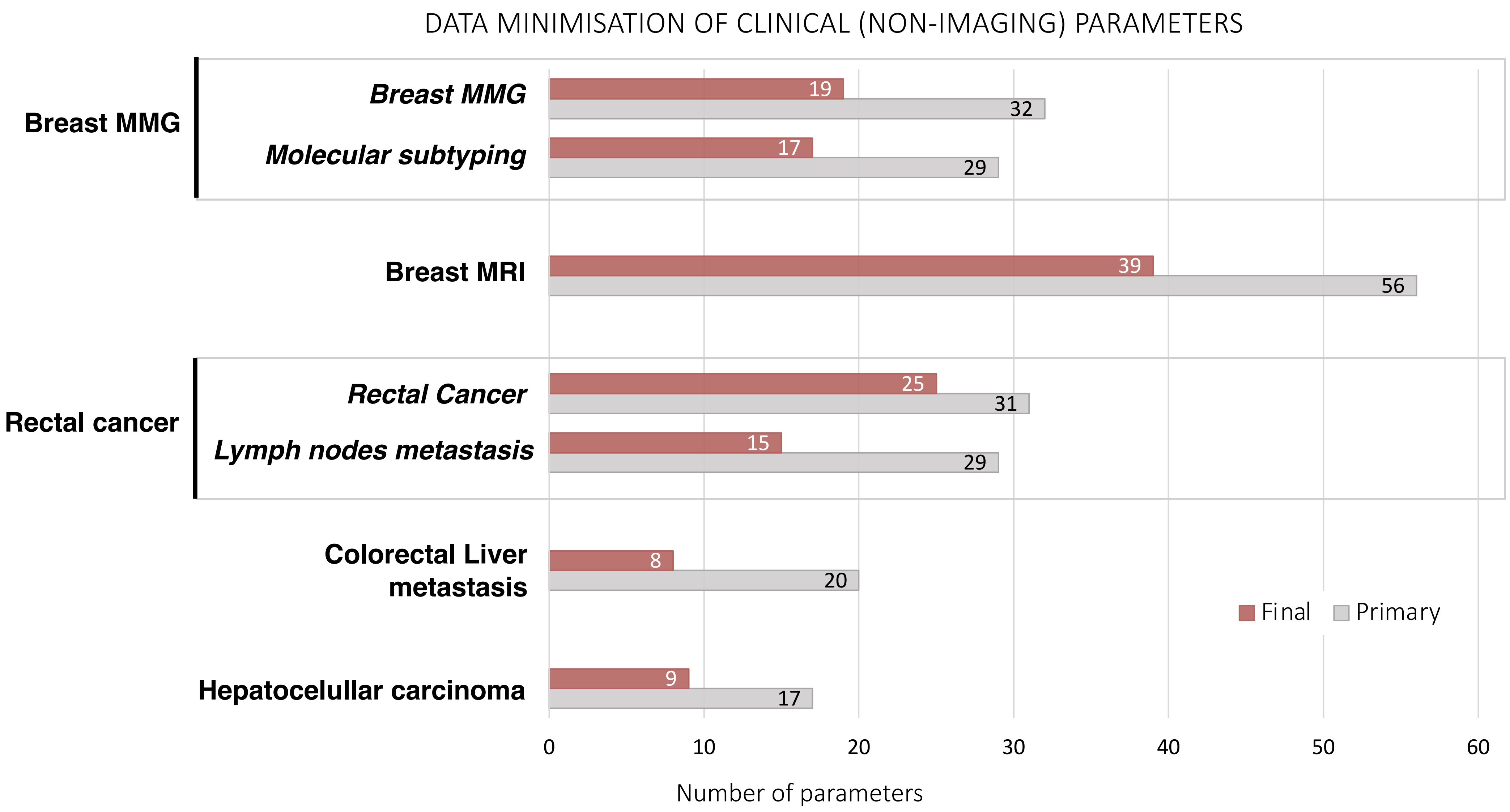

Click here to access/download;Figure;Figure 2_20240216.pdf

FIGURE 2

a

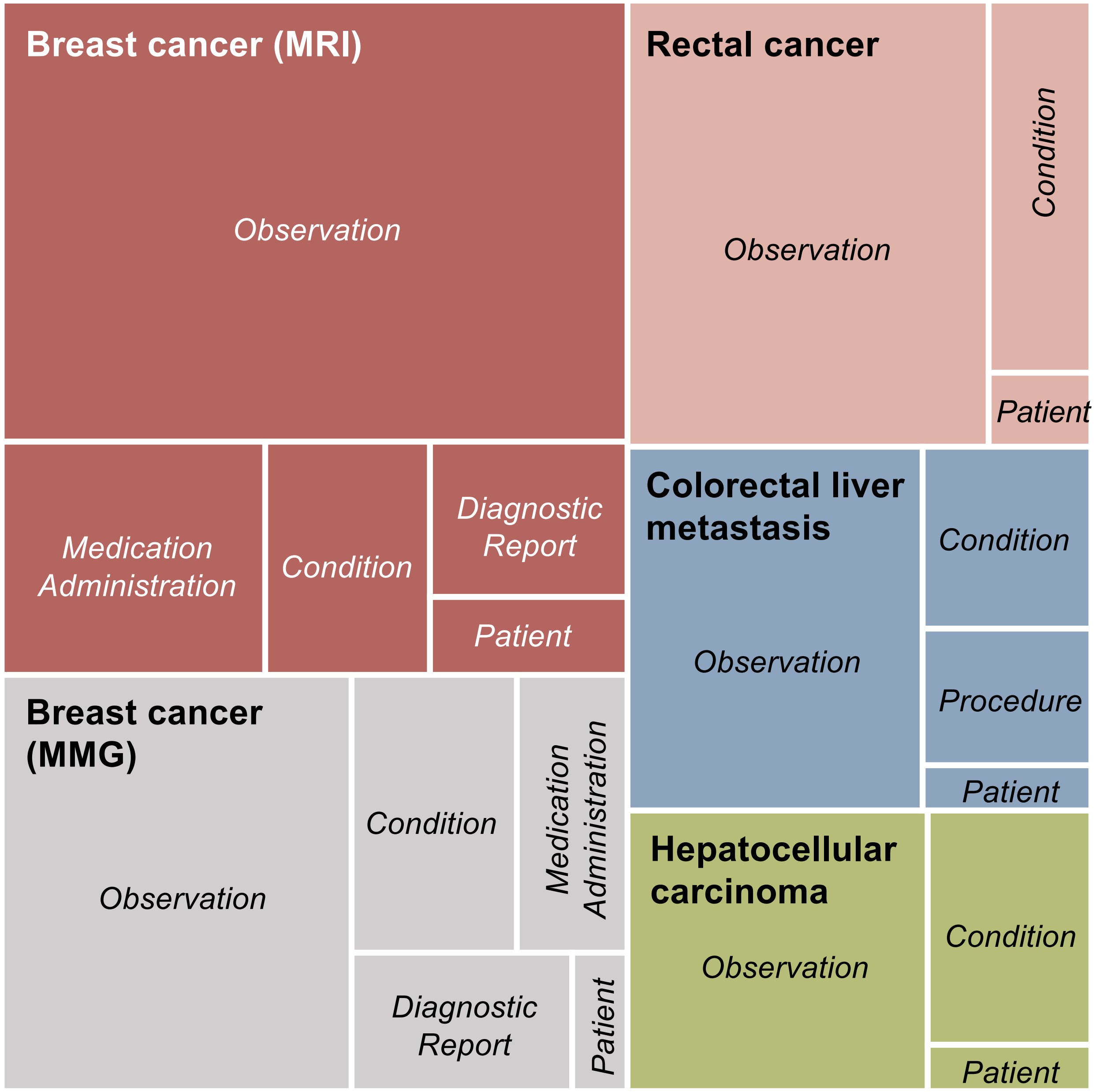

b

TERMONOLOGY	
RESOURCE: Patient	
Patient ID	
RESOURCE: Condition	
Condition – code<br>*For histopathology*	SNOMED<br>ICD-O3 (preferred) / SNOMED
Condition - clinical status	HL7/FHIR terminology
Condition – body site	SNOMED
RESOURCE: Observation	
Observation - code	SNOMED (preferred) / LOINC / NCIt
Observation - value	SNOMED (preferred) / LOINC / NCIt
RESOURCE: Procedure	
Procedure - code	SNOMED
Procedure - status	HL7/FHIR terminology
RESOURCE: Medication Administration	
Medication administrator - status	HL7/FHIR terminology
Medication administrator - medication	RxNorm (preferred) / SNOMED
Medication administration - dose	For units: UCUM
RESOURCE: Diagnostic report	
Diagnostic report - code	LOINC (preferred) / SNOMED
Diagnostic report - conclusion code	SNOMED

Click here to access/download;Figure;Figure 3_20240216.pdf

FIGURE 3

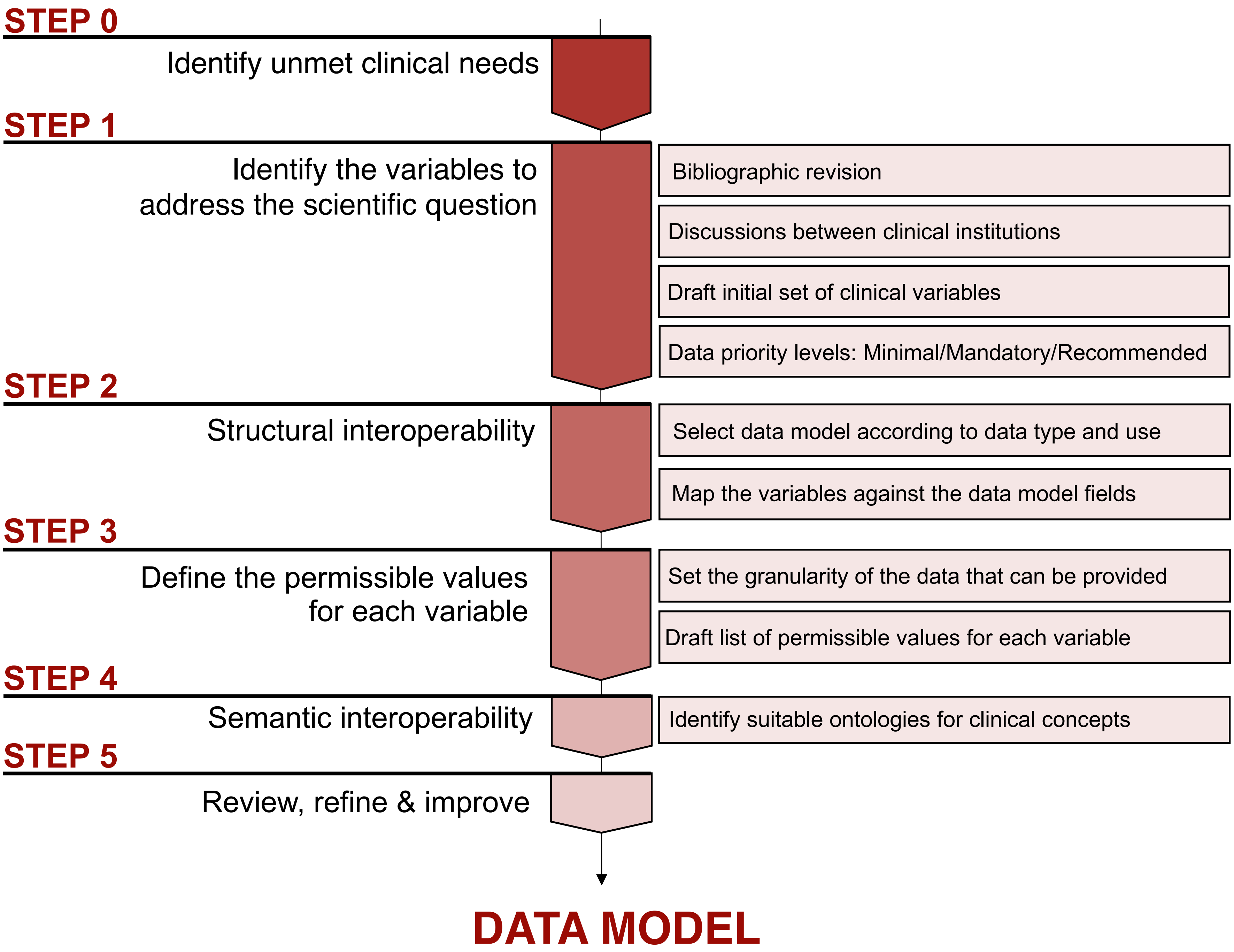

Figure 4

Click here to access/download;Figure;Figure 4_20240216.pdf

FIGURE 4

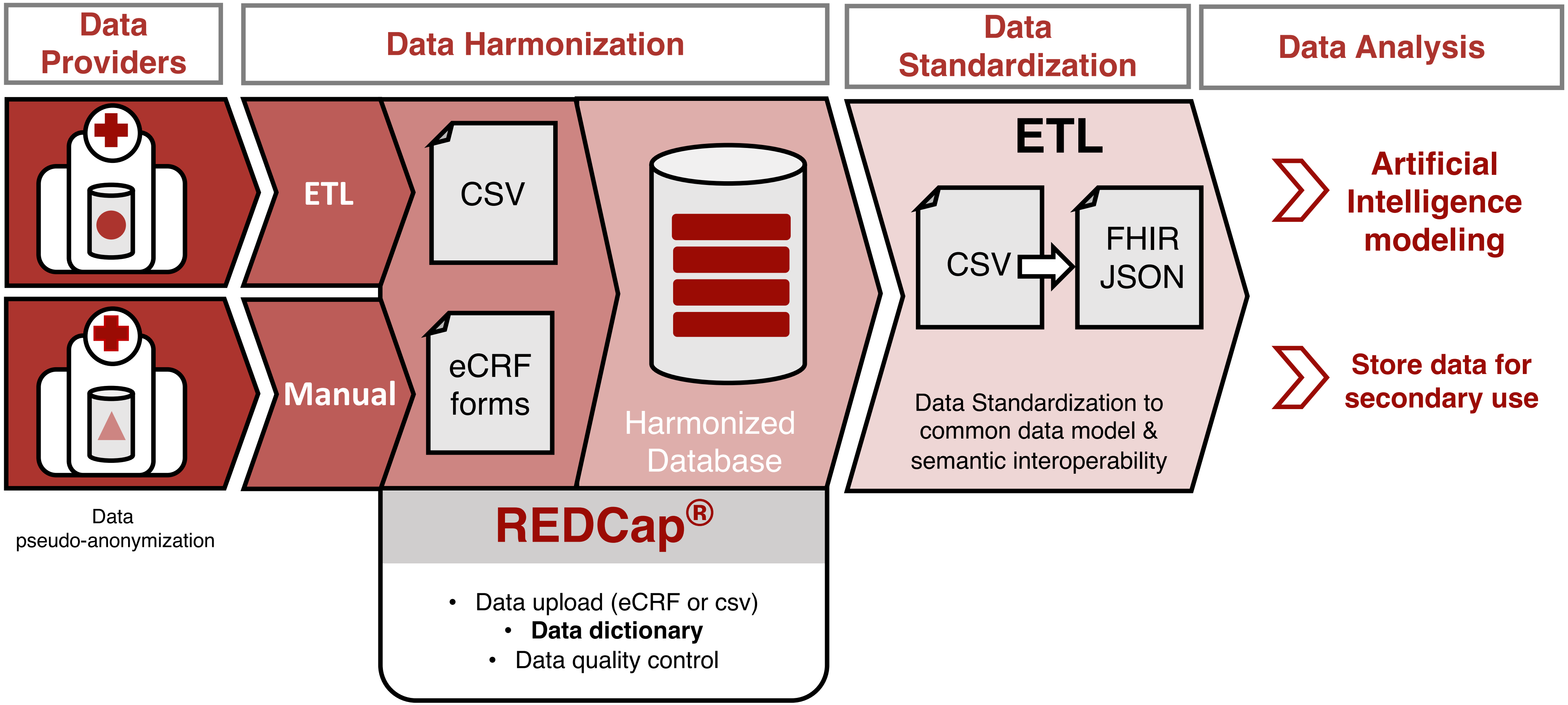

Supplement: giae101_GIGA-D-24-00085_Revision_1 [file giae101_giga-d-24-00085_revision_1.pdf]
