# Supplementary material for: New implementation of data standards for AI in oncology: Experience from the EuCanImage project
Source: Gigascience. 2025 May 13;14:giae101. doi: 10.1093/gigascience/giae101 (PMC12071370; doi:10.1093/gigascience/giae101)
Supplement: giae101_Authors_Response_To_Reviewer_Comments_Original_Submission [file giae101_authors_response_to_reviewer_comments_original_submission.pdf]

# Author's Response To Reviewer Comments

R1 - COMMENT 1
1. p.6: The authors mention "seven key unmet clinical needs in cancer imaging", but it is not clear what those refer to. It would be helpful to provide details about those needs, and provide more details about the data being collected for the individual applications. Also, if possible, please cite a publication that describes EuCanImage project.

Thank you for pointing this out. We have included a brief description of the use cases (page 6 - Data description) and project (page 5 - Background) for better understanding. Also in response to comment 3. We don't have a dedicated publication describing the project, however detailed information about EuCanImage can be found in our webpage (https://eucanimage.eu/#about-project) and in publications by the AI4HI network:
Kondylakis, H., Kalokyri, V., Sfakianakis, S. et al. Data infrastructures for AI in medical imaging: a report on the experiences of five EU projects. Eur Radiol Exp 7, 20 (2023). https://doi.org/10.1186/s41747-023-00336-x
Marti-Bonmati, L., Koh, DM., Riklund, K. et al. Considerations for artificial intelligence clinical impact in oncologic imaging: an AI4HI position paper. Insights Imaging 13, 89 (2022). https://doi.org/10.1186/s13244-022-01220-9
Kondylakis, H., Ciarrocchi, E., Cerda-Alberich, L. et al. Position of the AI for Health Imaging (AI4HI) network on metadata models for imaging biobanks. Eur Radiol Exp 6, 29 (2022). https://doi.org/10.1186/s41747-022-00281-1

R1 - COMMENT 2
2. The authors discuss in general terms the clinical use cases and cancers that are of interest for the data collection efforts, and mention some of the variables, but the complete list of the variables collected is not provided. Given that based on the Fig.1 the number of these variables is not exceedingly large, it would be appropriate to provide the complete list as an attachment to the manuscript.

We conceived this manuscript from a technical perspective (as technical note) and we considered that the description of the variables would benefit from an in-depth analysis of the clinical rationale behind that selection. The medical partners of the consortium are working on a specific manuscript with a clinical scope where we will provide a more detailed description of the variables, values and their clinical relevance. However, for technical usage, a basic list of the variables and the ontological codes used can be obtained within the script available in the github page:
https://github.com/EGA-archive/EuCanImage-FHIR/tree/main.

R1 - COMMENT 3
3. It would be rather interesting and important to better understand the use cases for utilization of the data collected.
* What are the operations that are expected to be performed on that data?

As suggested, for better understanding, we have included a more detailed description of the use cases (page 6 - Data description). The clinical data that is mentioned in this manuscript was collected as supporting information to complement the medical images, it contains the ground truth and serves as key supporting information to stratify patients into matched training or validation cohorts. It is, as well, additional data to be included as input in the AI models together with imaging data. The AI models try to respond the following questions:
-Hepatocellular carcinoma: Can AI increase the diagnostic sensitivity of liver MRI, currently at 60%, for detecting small HCC lesions (less than 20 mm) while keeping the specificity high?
-Colorectal liver metastasis: Can AI identify liver metastases in colorectal cancer from pre- and post-operative CT?
-Rectal cancer: Can AI predict the level of response to neoadjuvant radio(chemo)therapy based on primary MRI in rectal cancer for local staging and restaging?
-Rectal cancer - Lymph nodes metastasis: Can AI identify mesorectal lymph node metastases in pelvic

MRI?
-Breast MRI: Could AI tools enable to de-escalate neoadjuvant systemic therapy (NST) in patients highly likely to achieve a pathological complete response (pCR)?
-Breast MMG: Can AI improve the assessment of screening mammograms by automatically differentiating benign from malignant lesions?
-Breast MMG - Molecular subtyping: Can AI distinguish five molecular subtypes of invasive ductal breast carcinoma on MMG?

*Who is the target user for this dataset?

The target users for this datasets are first, AI developers within EuCanImage, and then other AI researchers who would be interested in re-using this curated data, once the data is available in the permanent repository and after data access approval.

*What are the key capabilities that become possible due to adoption of FHIR for data representation? This would be appropriate in order to justify the selection of FHIR for the representation of the harmonization result.

The adoption of FHIR enables syntactic and semantic interoperability, making the confluence with other EU initiatives easier and thus providing more scalability. At the beginning of the project we evaluated some potentially suitable data standards OMOP, ICGC-ARGO and FHIR. In EuCanImage, the data modeling needed to serve two main purposes: a) harmonization and standardization of the data for unambiguous meaning and b) secondary use by being available under request in a repository, so, being in a format adequate for distribution. FHIR outperformed the other standards that were evaluated for the representation of concepts for this clinical domain and presented significant advantages for sustainable storage and data sharing at the permanent repository, EGA. The rationale has been included in page 9 - Data Model Design and in response to reviewer 2 comment 15.

*Examples/use cases of what is possible now that was not possible before would be beneficial.

This workflow (from raw data to the storage of standardized data at EGA) provides a new approach to integrate and share data using different already existing components from for data capture (REDCap) to data standardization (FHIR) and storage in an existing permanent repository (EGA), together with new developed scripts to fill the gaps (ETL script), which, all together, favors the sustainability of the project and data FAIRness. Without this workflow we couldn’t have used the data from the different hospitals due to non-compatible data format and unstandardized terminology.

R1- COMMENT 4
4. Related to point 2 above, since the authors developed the dictionary and the value sets for the variables being harmonized, it would be highly relevant to include those dictionaries and value sets as an attachment/appendix to the manuscript.

See response to comment 2

R1 - COMMENT 5
5. It appears that the effort of mapping the site-specific variables is primarily done at the time data is collected into CSV or is submitted using the online forms. It is not entirely clear what is the effort for such harmonization, what is the logistics of this harmonization (ie, is this performed entirely by the site personnel or there is a central helpdesk that consults submitters on any questions arising). Further, given that the sites are located in different countries with different main languages, it would be helpful if authors could comment on the language barrier or the absence of such while collecting, harmonizing and submitting the data.

Thank you for pinpointing this, we have included additional insights to better describe the process in the revised manuscript (page 11 - Technical implementation of data standards and Figure 4 legend). Briefly, the harmonization efforts vary a lot between centers and use cases. Some sites had structured repositories with variables linked to standard terminologies that required minimal mapping and transformation efforts,

while others performed this task manually or through dictionaries that mapped local concepts to standard ones. In all cases, this was performed by site personnel, but with guidance from the Data Model Working Group of EuCanImage. Regarding the language barrier, many of the concepts from the terminologies that were used have multilingual descriptions, which reduced language barriers. In the rest of the cases, mappings were performed by personnel who understood the meaning of standard terms in English and their local equivalence.

R1 - COMMENT 6
6. Since the overall focus of the EuCanImage is on radiological data, and since presumably such data would be longitudinal, it is important to provide details on how de-identification related activities are coordinated with the collection of clinical data, and how key attributes of the data (ie, various identifiers and dates) are kept in sync between different data types.

We described the key de-identification methods we specifically used for the clinical data, basically a) the use of EuCanImage ID to codify patient IDs (page 12) and 2) the de-identification of dates by using time periods or the use of arbitrary dates to mimic the Epoch Unix system (page 11). The imaging data required for the use cases is not longitudinal, for each patient we have only one set of images collected at one time point. The specific methods for the de-identification of medical images is out of the scope of this manuscript, however, imaging data de-identification tools and methods were described in detail and compared with other AI4HI network projects in a separate image de-identification manuscript currently under review.

R1 - COMMENT 7
7. While it provides interesting information, the section "Legal interoperability" seems to be somewhat disconnected from the rest of the narrative.

We agree with the reviewer's assessment. Although this section about the legal aspects is not directly related with the main focus of the manuscript (data standardization and interoperability challenges) we considered it to be of interest for the reader. In EuCanImage, ethico-legal discussions have played an important role from the beginning of the project and continue to be a recurring topic in similar consortia and initiatives. In addition to the GDPR, the European legal framework is increasingly populated with regulations and norms that directly relate to the scope of this paper. With this section our aim is to provide an overview on legal and practical issues that we have identified during the project (e.g., a rapidly evolving regulatory environment, the anonymisation-pseudonymisation conundrum in the GDPR, legal barriers on secondary uses of personal data for AI research and development, cross-border data sharing etc.) and that are relevant when trying to implement data standards for data interoperability.

In response to the reviewer’s prescient feedback and for better integration and contextualization we have replaced the headers which could be misleading and included an introductory paragraph to better frame the purpose of this section and its coherence with the rest of the manuscript (pages 13-14).

Reviewer #2: This work can be of interest for researcher planning to develop similar data infrastructures.

From my point of view the main limitations of this work are:

R2- COMMENT 1
* The lack of discussion around the OMOP CDM

We appreciate the remark and extended the discussion around the rationale behind standards selection. See response to comment 15, also page 9 of the manuscript.

R2- COMMENT 2
* The lack of overview and details about the ETL process

We provided a more detailed description of the ETL. For further insights into this process, first we need to specify that we can refer to two different types of ELT pipelines in the project 1) from hospital raw data to RedCap and 2) from the harmonized RedCap database to the standardized FHIR compliant file. The first

ETL process was carried out directly by the clinical institutions with their own capabilities for doing an automatized data retrieval process. Description included in page 11. The second ETL process is common for all sites and it is performed centrally. For a more detailed description we added a specific section in methods (pages 18-19).

R2- COMMENT 3
* Limited reference to the FAIR data principles

We included a specific paragraph in the background (page 4). Interoperability is the focus of this work and we agree it was important to highlight the commitment of the project to the FAIR framework.

R2- COMMENT 4
* The claim that it is for precision oncology while it seems much more centered around imaging.

As the reviewer points out EuCanImage aims to build a platform around imaging data and the development of AI models in oncology. We appreciate the remark and for accuracy we have modified the wording, accordingly, throughout the document. See also comment 6.

Some more specific comments:

BACKGROUND
R2- COMMENT 5
* The reference 1 does not seem to back-up your claim that "Artificial intelligence (AI) for precision oncology is an exponentially growing field"

Thank you for pinpointing this out, we have updated the reference to:
Artificial Intelligence in Oncology: Current Landscape, Challenges, and Future Directions. William Lotter; Michael J. Hassett; Nikolaus Schult; Kenneth L. Kehl; Eliezer M. Van Allen ; Ethan Cerami. Cancer Discov (2024) 14 (5): 711–726. (https://doi.org/10.1158/2159-8290.CD-23-1199)

R2 - COMMENT 6
* Are we talking about precision medicine or precision oncology? And it is not very clear what you mean with precision oncology, typically is genomics based oncology, you should probably make it more explicit what you mean.

We agree with the perspective of the reviewer and we think this is a valuable appreciation, in accordance, we have refined the wording throughout the manuscript to be more explicit and accurate.

R2 - COMMENT 7
* I don't see your point about the broad implementation, typically data sharing is not for implementation but for research so I'm a bit confused here.

Thank you for pointing this out, we have simplified the sentence for concision (page 4). With implementation we refer to the process of translating the conceptual model we first developed (list of variables, the values, the codes…) into its actual application which also implies moving data between borders. Being sure that we were capable of fulfilling all the steps from data capture at hospitals, to data standardization and storage, testing that the concept was feasible using existing technologies when possible and creating custom scripts to cover the gaps when needed.

R2 - COMMENT 8
* "Generating and collecting the mentioned data types": all structured data can be stored as CSV, so the problem is to combine that with genomics (VCF?) and imaging (NIFTI?)

The text reads as follows: “Generating and collecting the mentioned data types involve a multitude of different technological platforms and their storage in a wide range of data formats and information systems, contributing to increased data diversity”. With this sentence we wanted to highlight the vast diversity of clinical data that is collected at healthcare institutions, which not only includes different data

types but also the different technological platforms that are used in the different institutions (ie: different vendors, versions…). All that diversity increases the complexity of the harmonization and standardization requirements. For clarity we have rephased specifying it refers to the diversity of technological platforms and storage systems among hospitals (data sources) not in other contexts.

R2 - COMMENT 9
* "cross-border legal constraints and massive data volumes required", I don't think this is related to interoperability, you should probably rephrase that part.

We see the point of the reviewer. Thank you for pinpointing this out. In accordance, we have deleted the sentence for clarity and concision (page 4).

R2 - COMMENT 10
* "such as the Health Level Seven (HL7®) or fast healthcare interoperability resources (FHIR®) specification", HL7 develops the FHIR standard, you should reprhase that.

Thank you for pinpointing this inaccuracy. We have corrected the sentence (page 4-5).

DATA DESCRIPTION
R2 - COMMENT 11
* You mention the 7 key unmet needs and I'm a bit struggling to find them, you should make it explicit where to find them.

We agree with the reviewer, we have specified the aim of the different use cases in the data description section (page 6). For additional details, the aims are also described in response to reviewer 1 comment 3.

R2 - COMMENT 12
* "The dialogue between physicians and AI developers on clinically relevant variables that can be meaningfully incorporated in AI algorithms, with the GDPR-compliant data minimisation principle, led to the final set of defined variables (Figure 1)." You should detail more this process, was it a consensus from 100 clinicians across Europe or 2 clinicians from the main center giving their opinion? Also the actual list would be of interest, you should put that in supplementary.

We appreciate the suggestion and we have included that information in page 7. Briefly, at the beginning of the EuCanImage project it was created a Clinical Working group encompassing clinical representatives from five clinical centers. Each clinical site delegated their specialists towards each use case. Specialists means radiologist, pathologist, clinical oncologist, surgical oncologist, radiotherapy specialist and the data managers. In general the Clinical Working Group meetings were attended by 10-20 doctors every two weeks for the first year of the project. After the general concepts of use cases were established, we separated breast, colorectal and liver subgroups of the Clinical Working Group into organ-related meetings with different specialists per use case and center. These organ-related specialized groups met every two weeks for the next six months to develop the final list of clinico-pathological variables that were deemed to provide ground truth and provide clinical data adding value to the imaging data in deep learning modeling. Regarding the final list of clinical variables, we considered that it would be essential to provide an adequate clinical context for the selection of the variables and delve into its clinical relevance. The medical partners of the consortium are working on a specific manuscript with a clinical scope containing the precise variables, therefore we would prefer not to disclose this final information at the moment if that's acceptable. However, for a technical perspective, a basic list of the variables and ontological codes used can be obtained in the script available on the GitHub page:
https://github.com/EGA-archive/EuCanImage-FHIR/tree/main.

R2 - COMMENT 13
* "between 8 and 39 parameters per use case", that sounds quite few for AI applications.

We understand the reviewer's point and have rephrased the text for clarification (page 7). The range between 8 and 39 parameters per use case only refers to the clinical attributes (what we call non-imaging

data). Those variables, among other key information, contain the ground truth and will be integrated with more parameters extracted from medical images (MRI, CT, or MMG) and DICOM metadata to generate the AI models.

R2 - COMMENT 14
* "Next, the value sets for each clinical variable were discussed in detail and defined to ensure understanding and a manageable range of values" what does it mean? That you constraine the values to a specific range where you cap the outliers? I feel the process could be better described.

Thank you for the suggestion. We have rephrased the sentence to include a more specific description of the process (page 7). Briefly, the values of certain variables were categorized into value sets in order to be able to standardize their representation and link such concepts with a terminological code.

DATA CURATION
Analysis of semantic interoperability and health standards
R2 - COMMENT 15
* You gloss over the OMOP CDM part while I believe this is a key point, why did not you chose to build upon that instead?

We carefully considered OMOP CDM as a potential standard to represent EuCanImage data. Both OMOP and FHIR allow the structuring and formatting of healthcare data, however they serve fairly different purposes. While OMOP is more oriented towards the representation (structure and content) of clinical data, FHIR is more focused on healthcare data exchange. In EuCanImage, the data modeling needed to serve two main purposes: a) harmonization and standardization of the data for unambiguous meaning and b) secondary use by being available under request in a repository, so, being in a format adequate for distribution. The decision of choosing FHIR over OMOP was not obvious from the beginning, one of the aspects we considered to make the decision was the data sharing potential. While OMOP presented good performance for data representation it supposed an additional challenge to meet our second aim, data sharing. One of the interesting points of EuCanImage is that the platform is built over existing repositories, favoring in a great measure its sustainability. For the re-use of clinical data EGA is the permanent repository where it will be stored, in this regard FHIR presented significant advantages for sustainable storage and sharing once the project ends. Additionally, the existence of FHIR to OMOP (and OMOP to FHIR) conversion could solve the interoperability issue between the two main schemas.

Data Model Design
R2 - COMMENT 16
* "we decided to use FHIR due to its wide adoption, flexibility and suitability for real-world data exchange." This is why I wouldn't call FHIR a CDM, it is too flexible.

In the previous point we elaborate the basis of our decision to use FHIR, the rationale is also included in page 9 of the manuscript.

R2 - COMMENT 17
* "Clinical elements necessary for each hypothesis were established by domain experts and interdisciplinary teams including clinicians and AI developers, considering different key data aspects" you need to explain that in more details, which key data aspects?

Thank you for the suggestion, we have further explained the important aspects for the selection of clinical elements in page 9. Briefly:
-Sample size
-Target population selection and characterization
-Clinical endpoints: pathological hallmarks, disease behavior, treatment response, patient prognosis…
-Type of outcome: binary, continuous, time to event
-Adequate ground truth
-Minimal amount of data principle
-Availability of specific variables at the data sources - for example in Sweden some cancer data comes from

the national registries not the hospital itself.

R2 - COMMENT 18
* "When choosing the most suitable resource for each selected variable, each resource's constraints were considered", you need to give an example

Thank you for the suggestion. We have included an example of a resource's selection based on related content and constraints when choosing a suitable FHIR Resource in page 9-10.

TECHNICAL IMPLEMENTATION OF DATA STANDARDS
R2 - COMMENT 19
* "To minimise the need to re-encode and simplify the data capture process, we created electronic case-report forms (eCRF) with REDCap." as you mention, this will require a lot for manual work that will be a big problem for scability and portability. It is a good tool for research but not that much for implementation as you are claiming the project is about.

We agree that data capture with eCRF forms is, in general, an intensive manual work and can be a problem for scalability. In this regard, there were differences in the capabilities of the different hospitals to extract data from EHR and to transfer into REDCap, while some centers used REDCap in a more classical manual way, others with more structured databases could automate the process with their own scripts. So, in some way, the extent of manual work was dependent on the capabilities of the different centers. In this workflow, REDCap was at the same time a tool for data capture and a common database used as a proxy for data harmonization. It is also important to specify that in this context, implementation refers to the translation of the plan (the conceptual model) to its execution, regardless of the scale.

DISCUSSION
R2 - COMMENT 20
* You claim to focus on interoperability but only mention the FAIR data principles at this stage. And there are already some litterature specifically about the FAIR data principles in precision oncology (for example DOI: 10.1093/bib/bbz044), it is probably a good idea to include that.

Thank you for the suggestion, we agree with the reviewer. Interoperability is a key part of the work and FAIR principles are a pillar of the project. We have included the suggested reference and spotlight the importance of the adherence to FAIR principles in the background (page 4).

R2 - COMMENT 21
* "The standardised datasets that we will make available" You need to explain when it will be available.

Data will be available under request approval at the end of the project. A sentence has been added for concretion.

Editorial Manager®

## Cookie Preference Center

We use cookies which are necessary to make our site work. We may also use additional cookies to analyze, improve and personalize our content and your digital experience. For more information, see our Cookie Policy.

You may choose not to allow some types of cookies. However, blocking some types may impact your experience of our site and the services we are able to offer. See the different category headings below to find out more or change your settings.

Allow all

## Manage Consent Preferences

#### Strictly Necessary Cookies

**Always active**

These cookies are necessary for the website to function and cannot be switched off in our systems. They are usually only set in response to actions made by you which amount to a request for services, such as setting your privacy preferences, logging in or filling in forms. You can set your browser to block or alert you about these cookies, but some parts of the site will not then work. These cookies do not store any personally identifiable information.

#### Functional Cookies

These cookies enable the website to provide enhanced functionality and personalisation. They may be set by us or by third party providers whose services we have added to our pages. If you do not allow these cookies then some or all of these services may not function properly.

#### Performance Cookies

These cookies allow us to count visits and traffic sources so we can measure and improve the performance of our site. They help us to know which pages are the most and least popular and see how visitors move around the site.

#### Targeting Cookies

These cookies may be set through our site by our advertising partners. They may be used by those companies to build a profile of your interests and show you relevant adverts on other sites. If you do not allow these cookies, you will experience less targeted advertising.

### Cookie List

Clear

Apply Cancel

Consent Leg.Interest

Confirm my choices

Powered by onetrus
