# Supplementary material for: New implementation of data standards for AI in oncology: Experience from the EuCanImage project
Source: Gigascience. 2025 May 13;14:giae101. doi: 10.1093/gigascience/giae101 (PMC12071370; doi:10.1093/gigascience/giae101)
Supplement: giae101_Authors_Response_To_Reviewer_Comments_Revision_1 [file giae101_authors_response_to_reviewer_comments_revision_1.pdf]

Author's Response To Reviewer Comments

Reviewer reports:
Reviewer #1: The revised manuscript is significantly improved, I appreciate the efforts of the authors.

R1 comment 1
- please cite the references included in the response in the revised manuscript; right now they do not appear to be cited
Thank you for pointing this out. The references included in the response have been added to the manuscript. Ref 12-14 page 5 and 6.
R1 comment 4
- I understand that the mapping is included in the referenced GitHub repository. I believe the manuscript should stand out on its own, and I recommend the authors consider including the mapped terms as an appendix to the manuscript.
We have included the mapping terms in the Github page, each use case contains its own machine compatible dictionary.

Language
- authors are encouraged to review the language to correct some grammar issues (e.g., "softwares", "setted up", break down some exceedingly long sentences to improve readability, correct spelling of the "Chaimilion" -> Chaimeleon?)
Thank you for the suggestion. We have corrected the grammar of the text.

Reviewer #2: The authors addressed adequatly my comments.

Editorial Manager®

Cookie Preference Center

We use cookies which are necessary to make our site work. We may also use additional cookies to analyze, improve and personalize our content and your digital experience. For more information, see our Cookie Policy.

You may choose not to allow some types of cookies. However, blocking some types may impact your experience of our site and the services we are able to offer. See the different category headings below to find out more or change your settings.

Allow all
Manage Consent Preferences

Strictly Necessary Cookies

Always active

These cookies are necessary for the website to function and cannot be switched off in our systems. They are usually only set in response to actions made by you which amount to a request for services, such as setting your privacy preferences, logging in or filling in forms. You can set your browser to block or alert you about these cookies, but some parts of the site will not then work. These cookies do not store any personally identifiable information.

Functional Cookies

These cookies enable the website to provide enhanced functionality and personalisation. They may be set by us or by third party providers whose services we have added to our pages. If you do not allow these cookies then some or all of these services may not function properly.

Performance Cookies

These cookies allow us to count visits and traffic sources so we can measure and improve the performance of our site. They help us to know which pages are the most and least popular and see how visitors move around the site.

Targeting Cookies

These cookies may be set through our site by our advertising partners. They may be used by those companies to build a profile of your interests and show you relevant adverts on other sites. If you do not allow these cookies, you will experience less targeted advertising.

Cookie List

Clear

Apply Cancel
Consent Leg.Interest

Confirm my choices

Powered by onetrust
