# Supplementary material for: New implementation of data standards for AI in oncology: Experience from the EuCanImage project
Source: Gigascience. 2025 May 13;14:giae101. doi: 10.1093/gigascience/giae101 (PMC12071370; doi:10.1093/gigascience/giae101)
Supplement: giae101_Reviewer_1_Report_Original_Submission — Andrey Fedorov -- 5/12/2024 [file giae101_reviewer_1_report_original_submission.pdf]

Reviewer Report

Title: New implementation of data standards for AI in oncology. Experience from the EuCanImage project.

Version: Original Submission **Date:** 5/12/2024

Reviewer name: Andrey Fedorov

Reviewer Comments to Author:

In this manuscript the authors introduce their approach for collecting clinical data in the context of a large multi-national EuCanImage data collection effort. The authors discuss their use of FHIR as a uniform mechanism for representing the clinical data attributes, and their approach to harmonize individual contributions, integrate various data collection tools and conversion of the collected content into FHIR resources. Overall, this is a rather valuable manuscript as it discusses the procedures implemented for an important data collection initiative and can serve as a resource for both understanding the implemented procedures and as a learning resource for similar efforts. The comments for improvement of the manuscript are mostly concerned with the clarifications and details about the work performed:1. p.6: The authors mention "seven key unmet clinical needs in cancer imaging", but it is not clear what those refer to. It would be helpful to provide details about those needs, and provide more details about the data being collected for the individual applications. Also, if possible, please cite a publication that describes EuCanImage project.2. The authors discuss in general terms the clinical use cases and cancers that are of interest for the data collection efforts, and mention some of the variables, but the complete list of the variables collected is not provided. Given that based on the Fig.1 the number of these variables is not exceedingly large, it would be appropriate to provide the complete list as an attachment to the manuscript.3. It would be rather interesting and important to better understand the use cases for utilization of the data collected. What are the operations that are expected to be performed on that data? Who is the target user for this dataset? What are the key capabilities that become possible due to adoption of FHIR for data representation? This would be appropriate in order to justify the selection of FHIR for the representation of the harmonization result. Examples/use cases of what is possible now that was not possible before would be beneficial.4. Related to point 2 above, since the authors developed the dictionary and the value sets for the variables being harmonized, it would be highly relevant to include those dictionaries and value sets as an attachment/appendix to the manuscript.5. It appears that the effort of mapping the site-specific variables is primarily done at the time data is collected into CSV or is submitted using the online forms. It is not entirely clear what is the effort for such harmonization, what is the logistics of this harmonization (ie, is this performed entirely by the site personnel or there is a central helpdesk that consults submitters on any questions arising). Further, given that the sites are located in different countries with different main languages, it would be helpful if authors could comment on the language barrier or the absence of such while collecting, harmonizing and submitting the data.6. Since the overall focus of the EuCanImage is on radiological data, and since presumably such data would be longitudinal, it is important to provide details on how de-identification related activities are coordinated with the

collection of clinical data, and how key attributes of the data (ie, various identifiers and dates) are kept in sync between different data types.7. While it provides interesting information, the section "Legal interoperability" seems to be somewhat disconnected from the rest of the narrative.

Level of Interest

Please indicate how interesting you found the manuscript: Choose an item.

Quality of Written English

Please indicate the quality of language in the manuscript: Choose an item.

Declaration of Competing Interests

Please complete a declaration of competing interests, considering the following questions:

- Have you in the past five years received reimbursements, fees, funding, or salary from an organisation that may in any way gain or lose financially from the publication of this manuscript, either now or in the future?
- Do you hold any stocks or shares in an organisation that may in any way gain or lose financially from the publication of this manuscript, either now or in the future?
- Do you hold or are you currently applying for any patents relating to the content of the manuscript?
- Have you received reimbursements, fees, funding, or salary from an organization that holds or has applied for patents relating to the content of the manuscript?
- Do you have any other financial competing interests?
- Do you have any non-financial competing interests in relation to this paper?

If you can answer no to all of the above, write 'I declare that I have no competing interests' below. If your reply is yes to any, please give details below.

I declare that I have no competing interests

I agree to the open peer review policy of the journal. I understand that my name will be included on my report to the authors and, if the manuscript is accepted for publication, my named report including any attachments I upload will be posted on the website along with the authors' responses. I agree for my report to be made available under an Open Access Creative Commons CC-BY license (http://creativecommons.org/licenses/by/4.0/). I understand that any comments which I do not wish to be included in my named report can be included as confidential comments to the editors, which will not be published.

Choose an item.

To further support our reviewers, we have joined with Publons, where you can gain additional credit to further highlight your hard work (see: https://publons.com/journal/530/gigascience). On publication of this paper, your review will be automatically added to Publons, you can then choose whether or not to claim your Publons credit. I understand this statement.

Yes Choose an item.
