# Supplementary material for: New implementation of data standards for AI in oncology: Experience from the EuCanImage project
Source: Gigascience. 2025 May 13;14:giae101. doi: 10.1093/gigascience/giae101 (PMC12071370; doi:10.1093/gigascience/giae101)
Supplement: giae101_Reviewer_2_Report_Original_Submission — Charles Vesteghem -- 5/16/2024 [file giae101_reviewer_2_report_original_submission.pdf]

Reviewer Report

Title: New implementation of data standards for AI in oncology. Experience from the EuCanImage project.

Version: Original Submission **Date:** 5/16/2024

Reviewer name: Charles Vesteghem

Reviewer Comments to Author:

This work can be of interest for researcher planning to develop similar data infrastructures.From my point of view the main limitations of this work are:* The lack of discussion around the OMOP CDM* The lack of overview and details about the ETL process* Limited reference to the FAIR data principles* The claim that it is for precision oncology while it seems much more centered around imaging.Some more specific comments:BACKGROUND* The reference 1 does not seem to back-up your claim that "Artificial intelligence (AI) for precision oncology is an exponentially growing field"* Are we talking about precision medicine or precision oncology? And it is not very clear what you mean with precision oncology, typically is genomics based oncology, you should probably make it more explicit what you mean.* I don't see your point about the broad implementation, typically data sharing is not for implementation but for research so I'm a bit confused here.* "Generating and collecting the mentioned data types": all structured data can be stored as CSV, so the problem is to combine that with genomics (VCF?) and imaging (NIFTI?)* "cross-border legal constraints and massive data volumes required", I don't think this is related to interoperability, you should probably rephrase that part.* "such as the Health Level Seven (HL7Â®) or fast healthcare interoperability resources (FHIRÂ®) specification", HL7 develops the FHIR standard, you should reprhase that.DATA DESCRIPTION* You mention the 7 key unmet needs and I'm a bit struggling to find them, you should make it explicit where to find them.* "The dialogue between physicians and AI developers on clinically relevant variables that can be meaningfully incorporated in AI algorithms, with the GDPR-compliant data minimisation principle, led to the final set of defined variables (Figure 1)." You should detail more this process, was it a consensus from 100 clinicians across Europe or 2 clinicians from the main center giving their opinion? Also the actual list would be of interest, you should put that in supplementary.* "between 8 and 39 parameters per use case", that sounds quite few for AI applications.* "Next, the value sets for each clinical variable were discussed in detail and defined to ensure understanding and a manageable range of values" what does it mean? That you constraine the values to a specific range where you cap the outliers? I feel the process could be better described.DATA CURATIONAnalysis of semantic interoperability and health standards* You gloss over the OMOP CDM part while I believe this is a key point, why did not you chose to build upon that instead?Data Model Design* "we decided to use FHIR due to its wide adoption, flexibility and suitability for real-world data exchange." This is why I wouldn't call FHIR a CDM, it is too flexible.* "Clinical elements necessary for each hypothesis were established by domain experts and interdisciplinary teams including clinicians and AI developers, considering different key data aspects" you need to explain that in more details, which key data aspects?* "When choosing the most suitable resource for each selected variable, each resource's constraints were considered", you need to give an exampleTECHNICAL

IMPLEMENTATION OF DATA STANDARDS* "To minimise the need to re-encode and simplify the data capture process, we created electronic case-report forms (eCRF) with REDCap." as you mention, this will require a lot for manual work that will be a big problem for scability and portability. It is a good tool for research but not that much for implementation as you are claiming the project is about.DISCUSSION* You claim to focus on interoperability but only mention the FAIR data principles at this stage. And there are already some litterature specifically about the FAIR data principles in precision oncology (for example DOI: 10.1093/bib/bbz044), it is probably a good idea to include that.* "The standardised datasets that we will make available" You need to explain when it will be available.

Level of Interest

Please indicate how interesting you found the manuscript: Choose an item.

Quality of Written English

Please indicate the quality of language in the manuscript: Choose an item.

Declaration of Competing Interests

Please complete a declaration of competing interests, considering the following questions:

- Have you in the past five years received reimbursements, fees, funding, or salary from an organisation that may in any way gain or lose financially from the publication of this manuscript, either now or in the future?
- Do you hold any stocks or shares in an organisation that may in any way gain or lose financially from the publication of this manuscript, either now or in the future?
- Do you hold or are you currently applying for any patents relating to the content of the manuscript?
- Have you received reimbursements, fees, funding, or salary from an organization that holds or has applied for patents relating to the content of the manuscript?
- Do you have any other financial competing interests?
- Do you have any non-financial competing interests in relation to this paper?

If you can answer no to all of the above, write 'I declare that I have no competing interests' below. If your reply is yes to any, please give details below.

I declare that I have no competing interests

I agree to the open peer review policy of the journal. I understand that my name will be included on my report to the authors and, if the manuscript is accepted for publication, my named report including any attachments I upload will be posted on the website along with the authors' responses. I agree for my report to be made available under an Open Access Creative Commons CC-BY license

(http://creativecommons.org/licenses/by/4.0/). I understand that any comments which I do not wish to be included in my named report can be included as confidential comments to the editors, which will not be published.

Choose an item.

To further support our reviewers, we have joined with Publons, where you can gain additional credit to further highlight your hard work (see: https://publons.com/journal/530/gigascience). On publication of this paper, your review will be automatically added to Publons, you can then choose whether or not to claim your Publons credit. I understand this statement.

Yes Choose an item.
